# EcoHIV infection of mice establishes latent viral reservoirs in T cells and active viral reservoirs in macrophages that are sufficient for induction of neurocognitive impairment

**DOI:** 10.1371/journal.ppat.1007061

**Published:** 2018-06-07

**Authors:** Chao-Jiang Gu, Alejandra Borjabad, Eran Hadas, Jennifer Kelschenbach, Boe-Hyun Kim, Wei Chao, Ottavio Arancio, Jin Suh, Bruce Polsky, JoEllyn McMillan, Benson Edagwa, Howard E. Gendelman, Mary Jane Potash, David J. Volsky

**Affiliations:** 1 Department of Medicine, Icahn School of Medicine at Mount Sinai, New York, New York, United States of America; 2 Department of Pathology and Cell Biology, Columbia University Medical Center, New York, New York, United States of America; 3 Department of Medicine, St. Joseph’s Regional Medical Center, Paterson, New Jersey, United States of America; 4 Department of Medicine, NYU Winthrop Hospital, Mineola, New York, United States of America; 5 Department of Pharmacology and Experimental Neuroscience, University of Nebraska Medical Center, Omaha, Nebraska, United States of America; University of North Carolina at Chapel Hill, UNITED STATES

## Abstract

Suppression of HIV replication by antiretroviral therapy (ART) or host immunity can prevent AIDS but not other HIV-associated conditions including neurocognitive impairment (HIV-NCI). Pathogenesis in HIV-suppressed individuals has been attributed to reservoirs of latent-inducible virus in resting CD4^+^ T cells. Macrophages are persistently infected with HIV but their role as HIV reservoirs *in vivo* has not been fully explored. Here we show that infection of conventional mice with chimeric HIV, EcoHIV, reproduces physiological conditions for development of disease in people on ART including immunocompetence, stable suppression of HIV replication, persistence of integrated, replication-competent HIV in T cells and macrophages, and manifestation of learning and memory deficits in behavioral tests, termed here murine HIV-NCI. EcoHIV established latent reservoirs in CD4^+^ T lymphocytes in chronically-infected mice but could be induced by epigenetic modulators *ex vivo* and in mice. In contrast, macrophages expressed EcoHIV constitutively in mice for up to 16 months; murine leukemia virus (MLV), the donor of gp80 envelope in EcoHIV, did not infect macrophages. Both EcoHIV and MLV were found in brain tissue of infected mice but only EcoHIV induced NCI. Murine HIV-NCI was prevented by antiretroviral prophylaxis but once established neither persistent EcoHIV infection in mice nor NCI could be reversed by long-acting antiretroviral therapy. EcoHIV-infected, athymic mice were more permissive to virus replication in macrophages than were wild-type mice, suffered cognitive dysfunction, as well as increased numbers of monocytes and macrophages infiltrating the brain. Our results suggest an important role of HIV expressing macrophages in HIV neuropathogenesis in hosts with suppressed HIV replication.

## Author summary

Antiretroviral therapy (ART) transformed HIV infection from a death sentence to a chronic, largely manageable condition, with near preservation of immune systems and extended life expectancies in infected people. Despite effective ART, roughly half of HIV-infected people suffer problems in learning and memory that can impair day to day activities. In addition, HIV persists in largely silent states during ART, but it can be reactivated and produce infectious, possibly pathogenic virus. We addressed these two issues by infecting mice with a genetically engineered HIV that infects rodents instead of human beings. Unlike infected people we find that infected mice maintain good immune responses to HIV that appear to act like ART to keep virus levels low and allow mice to resist other complications. Like infected people, HIV-infected mice are subject to learning and memory problems; they carry silenced HIV long-term in T lymphocytes but we found that macrophages, infected in both humans and mice, continue to produce HIV in mice indicating that additional means may be needed to control HIV-infected macrophages. Moreover, in mice lacking T lymphocytes HIV infection of macrophages is sufficient to drive dysfunction in learning and memory. Like in people living with HIV, antiretroviral therapy in chronically-infected mice is ineffective against existing HIV infection and cognitive disease. Controlling this infection and brain disease may require tools tailored to actively expressed virus.

## Introduction

Combination antiretroviral therapy (ART) has changed the course of HIV pathogenesis. By stable suppression of virus replication, immune competence is maintained, serious AIDS-related conditions are largely prevented, and infected individuals live longer [1–3]. Despite these treatment benefits, HIV-suppressed patients remain at high risk of chronic diseases affecting gut, heart, and other tissues [4, 5]. In the central nervous system (CNS), ART shifted HIV neuropathogenesis from the severe cognitive, psychiatric, and motor defects of dementia to milder chronic forms of neurocognitive impairment (HIV-NCI) that can disturb performance of everyday tasks and worsen with age [6–8] (reviewed in [9, 10]]. Generally, chronic HIV morbidities are attributed to two factors, the persistence of replication competent HIV at low burdens within stable reservoirs of resting T lymphocytes [11, 12] and secondarily, metabolic effects of some prolonged antiretroviral treatment [13, 14]. How HIV contributes to individual chronic diseases in patients on ART is poorly understood.

We are interested in the role of HIV in NCI development under the physiological conditions of stable HIV suppression and functional immune systems, as seen in about 50% of patients on long-term ART [15–19]. HIV-NCI is also observed at significant frequencies in untreated individuals in relatively early stages of infection when antiviral responses suppress HIV replication prior to the onset of severe immunodeficiency [20–22]. Interestingly, the frequency of NCI in patients who initiated ART within one year on average after HIV transmission and in a matched cohort of untreated patients was similar [22, 23], suggesting that HIV depots driving NCI pathogenesis are established by the first year of HIV infection and these depots persist despite ART and vigorous host antiviral immune responses.

The consideration of HIV reservoirs in the context of NCI poses a conceptual challenge. HIV replicates in people in CD4^+^ T cells and cells of the monocyte lineage but it is generally accepted its major reservoir resides in latently infected resting CD4^+^ T cells [11, 12]. These reservoirs form early in infection [24] as productively-infected T cells are eliminated by HIV cytopathic effects and cytotoxic T cells (CTL) generated in response to HIV antigen expression [25]. The T cell reservoirs can be activated *ex vivo* to produce replication-competent virus [26] and are thought to contribute to residual viremia in HIV patients on ART by spontaneous reactivation *in vivo* [27]. Consistent with nervous system diseases caused by other lentiviruses however, HIV neuropathogenesis has been attributed to infection of myeloid cells including microglia [28–30]. At present, there is scant evidence of latent HIV in myeloid cells in hosts with stable ART-mediated HIV suppression. One study using laser microdissection and PCR found preponderance of HIV DNA-positive/HIV antigen negative perivascular macrophages, microglia, and astrocytes in brain tissues from five asymptomatic patients without NCI [31]. Others tested large number of mild NCI brain samples available through the National NeuroAIDS Tissue Consortium and found exceedingly low HIV DNA brain burdens in these patients [32, 33]; the cellular source of this DNA was not determined. A few studies suggested a link between HIV DNA burdens in circulating monocytes and cognitive impairment [34, 35]; and HIV expression and diversification has only recently been shown associated with tissue macrophages despite effective ART [36]. Chronically-infected macrophages are resistant to viral apoptosis, insensitive to post-infection ART, and relatively insensitive to antiviral CTL [37, 38] suggesting that these cells survive infection and can serve as HIV factories and promote mild NCI [39–41].

Here we probed the identity of HIV reservoirs and the development of NCI during experimental infection of mice by chimeric HIV, EcoHIV. EcoHIV was constructed using HIV NDK [42] with the replacement of the gp120 gene by ecotropic MLV gp80 gene for entry into cells through the widely expressed catamino acid transporter, CAT-1 [43, 44]. EcoHIV infection of mice is particularly suitable for posing these questions because we and others have shown that the virus can persistently infect conventional mice targeting CD4^+^ T cells, macrophages, and microglia [45–49]. Moreover, after an initial burst of virus replication, mice mount effective antiviral immune responses that limit HIV expression [47]. Despite EcoHIV suppression in these mice, the virus causes blood-brain barrier dysfunction [50], invades the brain at low levels [45, 48], and induces cytokine responses in the brain consistent with mild brain disease in humans [48, 51]. Here we show that mice persistently infected by EcoHIV remain immunocompetent, maintain stable/inducible HIV reservoirs in resting CD4^+^ T cells, carry constitutively expressed HIV in macrophages, and develop NCI detectable in two functionally independent learning/memory behavioral tests. T cell infection was not required for NCI in EcoHIV-infected athymic mice which express HIV extensively in macrophages and which show increased migration of monocytes and macrophages to the brain. Our data suggest an important role of macrophages persistently expressing HIV in NCI pathogenesis.

## Results

### The HIV genome integrates efficiently into mouse genomic DNA *in vivo*

**Fig 1:**
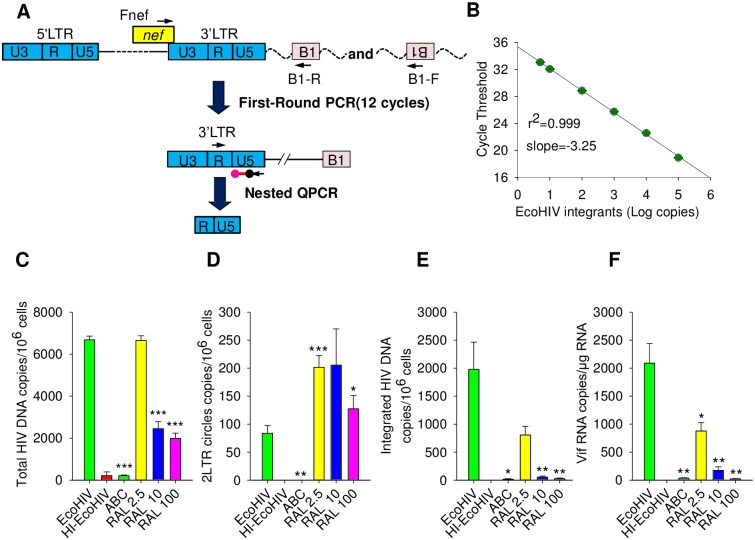
EcoHIV integrates efficiently in the murine host genome. **A.** Schematic representation of strategy for assay of integration. In the first round PCR, primers, B1-F and B1-R, from the host *B1* repetitive elements and a HIV-specific primer, Fnef, were used to amplify a region of integrated virus and the host flanking region. The second nested QPCR based on the HIV RU5 region was performed using the preamplified products. **B.** Integration standard curve was generated by logarithmic regression of the amplified IS sample signals. **C.-F.** Mice (n = 4/system) were pretreated with ABC or RAL 24 h before EcoHIV infection, euthanized 3 days later, and spleens collected for measurement of **C.** total, **D.** integrated, and **E.** 2-LTR circle vDNA; **F.** spliced vRNA by QPCR of DNA or cDNA as described in Methods.

It was previously reported that inefficient HIV DNA integration in mouse cell lines *in vitro* blocked HIV infection of mouse cells [52, 53]. Because EcoHIV can efficiently infect conventional mice [48, 50, 51, 54], we first determined the extent and specificity of EcoHIV DNA integration into the mouse genome. We employed QPCR as previously described [55] and modified methods for measurement of integrated HIV DNA [56]. The method employs a two-step PCR strategy using the most abundant of the mouse SINE elements, *B1* [57], as the target for amplification in the mouse genome (Fig 1A). To improve the efficiency of PCR amplification of the HIV-LTR-*B1* junction region, the first-round PCR amplification employed a sense LTR primer and two (sense and antisense) *B1* region PCR primers to reflect diverse integration sites [58] (Fig 1A). A clone of EcoHIV-infected Raw 248 cells served as a standard for integration. The standard curve constructed using the standard and primers has a dynamic range of over 4 logs and a detection limit of 5 integrated copies per reaction (Fig 1B). To test the specificity of the amplification strategy, mice were treated before and during infection with the nucleoside analogue reverse transcriptase inhibitor abacavir (ABC) or the integrase inhibitor raltegravir (RAL); 3 days after infection spleen cells were isolated, subjected to QPCR amplifying several forms of vDNA as well as spliced vRNA, the latter to demonstrate conclusively *de novo* HIV RNA synthesis [55] (Fig 1C–1F). *In vivo*, ABC inhibits all vDNA synthesis and consequently viral DNA integration and vRNA synthesis as well. In contrast, RAL inhibited in a dose-dependent manner only EcoHIV DNA integration and HIV RNA transcription (Fig 1E and 1F). As expected in a short-term infection, RAL only partially reduced total vDNA burden (Fig 1C) with about 30% of the detected PCR product likely representing the first-round vDNA synthesis from the virus inoculum. Additionally, RAL increased the number of copies of 2-LTR DNA (Fig 1D), presumably by raising the content of unintegrated nuclear DNA available for self-ligation, similar to the outcome of RAL intensification treatment in clinical trials [59]. These findings indicate that EcoHIV preserves HIV-integrase specific functions and that the frequency of EcoHIV integration in spleen cells during acute infection of mice is about 0.02% (Fig 1E), resembling that in CD4^+^ cells in patients with acute HIV infection [60].

### EcoHIV establishes persistent infection in mice without induction of immunodeficiency

**Fig 2:**
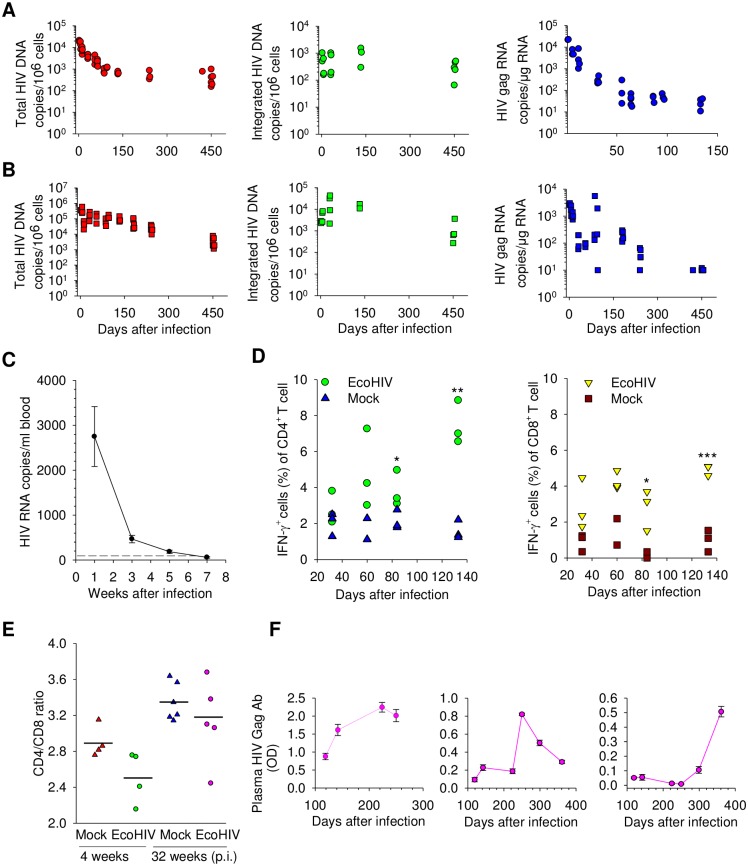
EcoHIV infects mice and induces antiviral immune responses but not immunodeficiency in mice. **A.-B.** Kinetics of production of total EcoHIV DNA (left panels), integrated EcoHIV DNA (middle panels), and genomic EcoHIV RNA (right panels) were measured by QPCR in **A.** spleen and **B.** PC at the times indicated after infection. Each point represents a single mouse. **C.** EcoHIV *gag* RNA burden at each time point in copies per ml of whole blood was measured at the times after infection indicated. The dashed line indicates limit of detection of 10 copies. **D.** The frequency of interferon-γ expression by CD4^+^ or CD8^+^ spleen cells at the time indicated after EcoHIV or mock infection was measured by flow cytometry. **E.** The number of CD4^+^ and CD8^+^ T cells was measured by flow cytometry at the indicated times and the CD4^+^:CD8^+^ ratio is shown. Symbols represent individual mice, the horizontal lines represent the mean. **F.** Longitudinal anti-NDK Gag antibodies in 3 EcoHIV-infected mice were measured by ELISA at the times indicated after infection. Each panel represents titers in a single mouse, mean +/- standard errors are shown.

The characteristics of EcoHIV infection of conventional mice that may be responsible for the observed HIV persistence and mild pathologies [45, 48, 50, 51] have not been fully elucidated. Here we determined the duration of HIV infection in mice over a period of 15 months characterizing the persistence of various forms of the viral genome, measuring some antiviral immune responses as well as the extent of immune competence (Fig 2 and S1 Fig). Groups of EcoHIV-infected mice were bled longitudinally and mice were euthanized in groups of 3–5 animals at time points indicated for virological analyses (Fig 2A, 2B and 2F). Measurement of virus burden over time revealed that after an early peak of virus replication and expression, EcoHIV achieves a plateau in spleen cells and peritoneal cells (PC), with integrated virus persisting in 0.02–0.1% of cells for the entire 15 months after infection (Fig 2A and 2B). As determined by integrated EcoHIV DNA and RNA loads, virus integration and expression continued in PC at higher levels than in spleen cells. In spleen, Gag RNA plateaus at roughly 50 copies per μg total RNA two months after infection, PC carry roughly 200 HIV RNA copies per μg total RNA up to six months after infection but virus expression at about 10 copies per μg (close to limit of detection) was still detectable in some animals 450 days after infection (Fig 2B, right panel). We observed transient EcoHIV viremia consistent with an early peak of HIV replication (Fig 2C). The control of EcoHIV expression in T cells in mice may be immunological in origin, including innate [45] and adaptive immune responses [47]. Here we confirm the latter finding by showing that CD4^+^ and CD8^+^ T cells mount interferon-γ responses to HIV Gag peptides over the course of chronic infection (Fig 2D, left and right panel). Of note, the T cell responses increased over time (80 and 140 days) suggesting both the continued expression of HIV antigens and continued immune competence of T cells during chronic infection in mice. Immune competence of HIV-infected animals was also indicated by stable CD4^+^:CD8^+^ cell ratios at 1 and 4 months after infection (Fig 2E) and induction of adaptive immune responses when infected mice were challenged with an unrelated antigen (S1 Fig). Finally, during long-term infection, individual mice demonstrated distinct patterns of anti-Gag antibodies, and by implication, stochastic fluctuations in Gag protein expression (Fig 2F). These findings indicate that EcoHIV establishes chronic infection in immunocompetent mice and does not induce immunodeficiency; in spleen cells the virus persists at low burdens mainly as silent integrated DNA from about 2 months after infection; virus silencing derives at least in part from host antiviral immunity [45, 47].

### Resting CD4^+^ T cells in chronically-infected mice serve as reservoirs of latent EcoHIV provirus which can be induced to expression and transmission by epigenetic modulators *in vitro* and *in vivo*

**Fig 3:**
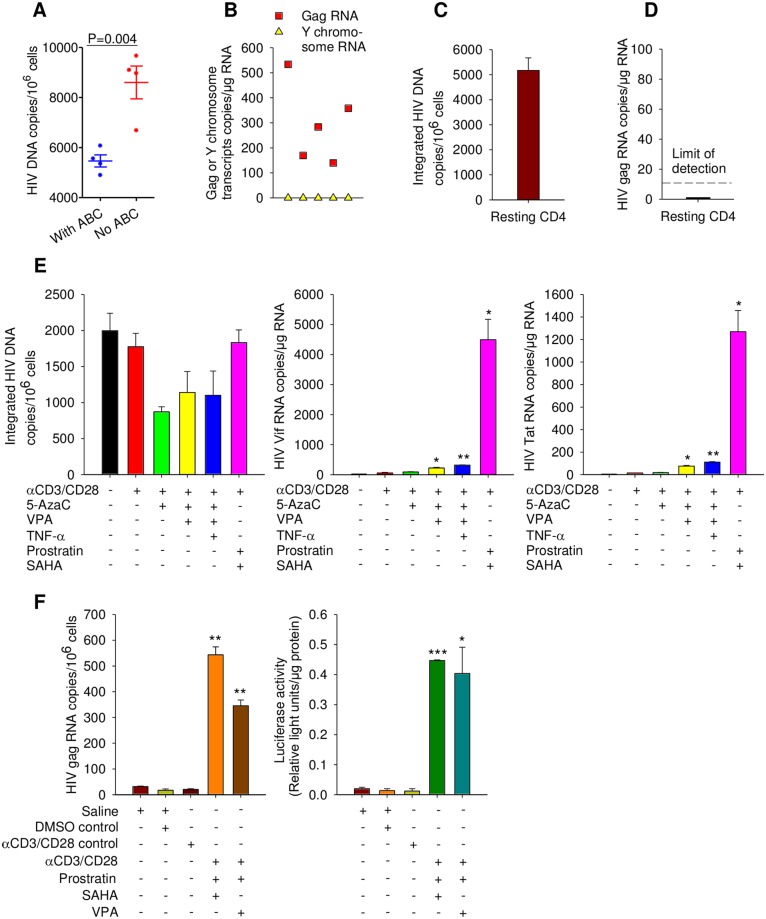
EcoHIV establishes latent reservoirs in resting CD4^+^ cells that are inducible by epigenetic modulators. **A.** CD4^+^ T cells isolated from spleen at day 25 post-infection were cocultured with uninfected cells in the presence or absence of ABC as indicated prior to vDNA amplification, each symbol represents culture from a single mouse. **B.** Six weeks after EcoHIV infection, CD4^+^ splenic T cells were harvested and purified from donor male 129X1/SvJ mice and injected into the recipient female 129X1 nude mice; their PM were collected after one week. Y chromosome and EcoHIV *gag* RNA levels in PM were determined by QPCR. Each symbol represents a single mouse. **C.-D.** Twelve weeks after infection, resting CD4^+^ T cells were purified from the spleen and **C.** The levels of integrated EcoHIV DNA and **D.** Genomic RNA were measured by QPCR. **E.** Eight weeks after EcoHIV infection of mice, resting CD4^+^ T cells were isolated and exposed to the agents indicated in culture for 2 days prior to collection and measurement of EcoHIV mRNA QPCR. **F.** Six weeks after infection by EcoHIV-Luc, mice were treated as shown, euthanized and splenic CD4^+^ cells isolated for measurement of virus RNA expression (left panel) or protein expression (right panel). *P<0.05, **P<0.01, ***P<0.001.

We previously demonstrated that EcoHIV infection in mouse spleen *in vivo* resides primarily in CD4^+^ T cells [48]. To determine whether EcoHIV provirus in these cells encodes infectious virus, two approaches were employed. First, 25 days after mouse infection, CD4^+^ cells were isolated from spleens and cocultured with CD8-depleted uninfected autologous spleen cells to allow virus spread, some cultures contained ABC, to distinguish provirus in donor cells from that in newly infected cells (Fig 3A). EcoHIV burden increased during culture with susceptible cells but culture in ABC significantly reduced vDNA burden, demonstrating that the increase observed represents new infection. Second, to investigate the *in vivo* infectivity of EcoHIV from CD4^+^ reservoirs, cells purified from male 129X1 mice 6 weeks after infection were transferred to athymic (nude) females of the same breed. One week after CD4^+^ cell transfer, EcoHIV burden in recipient PC was determined with an abundant, ubiquitous Y chromosome transcript measured in parallel to control for potential contamination by donor T cells (Fig 3B). EcoHIV RNA was detected in PC in each recipient with fewer than 20 male cells detected, indicating that female mouse PC were infected *in vivo* by progeny virus from infected male donor CD4^+^ T cells.

Because the primary latent HIV reservoir in infected human beings are resting CD4^+^ T cells [26], we next investigated the EcoHIV presence and expressional state in resting CD4^+^ cells of chronically-infected mice (Fig 3C and 3D). Resting CD4^+^/CD25^-^/CD69^-^ T cells were isolated from spleens 12 weeks after infection; flow cytometry analysis determined that CD4^+^ cells amounted to about 15% of the total spleen cells and the great majority of CD4^+^ T cells in this preparation were resting (low for CD25 and CD69) (S2 C). Determination of integrated EcoHIV DNA burden in these cells revealed approximately 5x10^3^ proviral DNA copies per 10^6^ cells (Fig 3C), representing 5-10-fold enrichment in this measure compared to unfractionated spleen cells and indicating that the majority of the integrated EcoHIV in chronic infection resides in resting CD4^+^ T cells. Despite moderate vDNA burden in these cells, no vRNA was detected (Fig 3D) indicating that resting CD4^+^ T cells serve as a reservoir of latent EcoHIV in chronically-infected mice, analogous to virus in PBMC from HIV-infected patients with suppressed HIV replication (S3 Fig, S1 Table) [24, 26].

Given that HIV provirus in resting human CD4^+^ T cells can be reactivated to virus production [61], we tested whether EcoHIV behaves similarly in chronically-infected mice. We evaluated several agents that have been shown to activate latent HIV in human cells: prostratin [62], valproic acid (VPA) [63], 5-azacytidine [64] and suberoylanilide hydroximic acid (SAHA) [65]. The agents were tested for their ability to induce EcoHIV *in vitro* in resting spleen CD4^+^ T cells isolated from chronically-infected ART naive mice (Fig 3E) or directly in chronically-infected mice followed by immediate analysis of spleen CD4^+^ T cells (Fig 3F). For induction *in vitro*, resting CD4^+^ T cells were isolated 8 weeks after EcoHIV infection and cultured in the presence of the agents indicated for two days (Fig 3E). We measured integrated EcoHIV DNA and its reactivation by measurement of *vif* and *tat* RNA (Fig 3, respective left to right panels). Consistent with data in Fig 3C and 3D, in the absence of any activation integrated EcoHIV DNA was detected in about 0.2% of cells and this provirus was transcriptionally silent (Fig 3E). Treatment of cells with T cell activating antibodies anti-CD3/anti-CD28 alone or in combination with DNA demethylation agent 5-azacytidine [64] failed to induce latent EcoHIV, but supplementing this combination with histone deacetylase inhibitor, VPA [63] or VPA and protein kinase C activator, prostratin [62] resulted in a small (10–20 fold) but significant provirus induction. In contrast, the combination of activating antibodies, prostratin, and epigenetic modulator SAHA [65] was by far the most efficient inducer of HIV RNA reaching 130-fold (for *tat* RNA) to 450-fold (for *vif* RNA) increase, a larger induction of reservoirs in CD4^+^ T cells than seen using SAHA for effects on cells from patients on effective ART [65] (Fig 3E). The latter combination appeared to be the least cytotoxic to induced cells as indicated by the preservation of the provirus burden (Fig 3E, left panel). To directly evaluate whether EcoHIV reservoirs in T cells *in vivo* can be induced by epigenetic modulators analogously to those in HIV-infected persons [63, 65], 6 weeks after infection mice were treated with different combinations of activating antibodies and epigenetic inducers for 2 days and then induction of vRNA in splenic CD4^+^ T cells was evaluated (Fig 3F, left panel). For assessment of viral protein induction *in vivo*, a similar experiment was conducted using EcoHIV encoding luciferase (S7 Fig) to allow convenient quantitation of its burden in mouse tissue (Fig 3F, right panel). Six weeks after infection, EcoHIV in splenic CD4^+^ T cells was effectively latent; however, mice receiving either the combination of mitogenic antibodies, prostratin, and SAHA effective *in vitro*, or this combination with VPA replacing SAHA, had CD4^+^ T cells with active EcoHIV transcription and protein expression (Fig 3F). Taking DMSO (vehicle) treatment as a baseline for latent HIV expression, the induction at provirus transcription was about 35-fold for VPA and 55-fold for SAHA with equivalent induction at the protein level (Fig 3F). Results from Fig 3 indicate that EcoHIV produced by infected CD4^+^ T cells is infectious (Fig 3A and 3B) and that following an early peak of expression, it assumes a latent, epigenetically inducible reservoir in these cells (Fig 3E and 3F).

### Macrophages persistently express EcoHIV in chronically-infected mice

**Fig 4:**
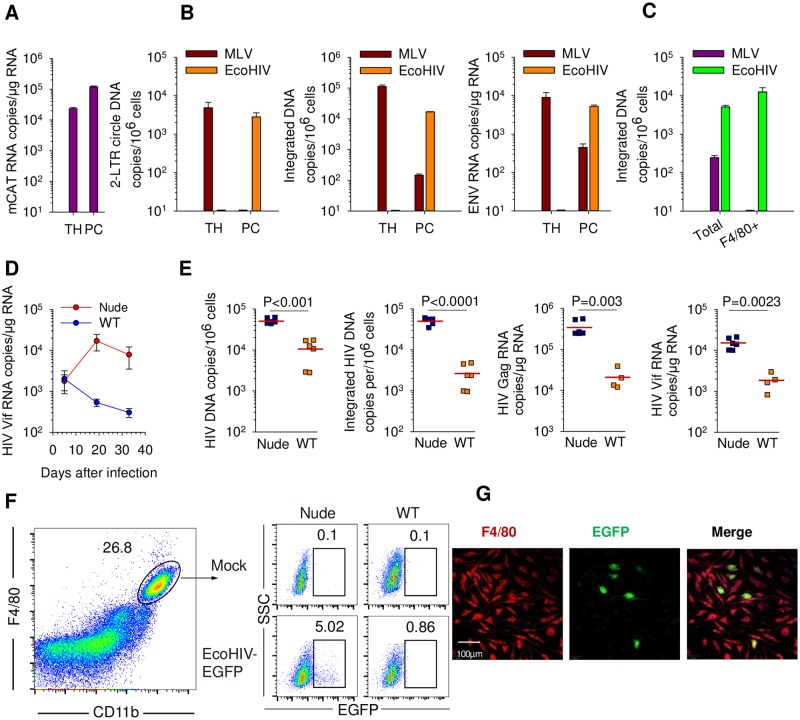
Mouse macrophages are susceptible to EcoHIV but not MLV. **A.** Expression of CAT-1 was determined in peritoneal cells (PC) and thymus (TH) of C57BL/6 mice by QPCR. **B.** Ten days after EcoHIV or MLV infection of mice, the indicated tissues were harvested for measurement of viral nucleic acids by QPCR. **C.** Eight weeks after mouse infection by EcoHIV or MLV, F4/80^+^ macrophages were purified from PC and integrated viral DNA in unfractionated or F4/80^+^ PC was measured by nested-QPCR. **D.** C57BL/6 euthymic and athymic mice were infected by EcoHIV and PC harvested from groups at the times indicated to measure spliced EcoHIV RNA. Mean and standard errors are shown (*P<0.05). **E.** At day 86 after EcoHIV infection, PC were isolated from 129X1 euthymic and athymic mice and forms of the viral DNA and RNA were measured by QPCR, each symbol represents a single mouse. **F.** Flow cytometric analysis of percentage of F4/80^+^ PM expressing EcoHIV-encoded EGFP in nude and wild-type mice at 25 days after infection. **G.** Expression of EcoHIV-encoded EGFP in F4/80^+^ PM at 25 days after infection in mice was detected by confocal imaging.

In addition to CD4^+^ T cells, macrophages are major target cells for HIV in human beings [66, 67]. Thus far our demonstration of EcoHIV infection in macrophages was limited to the mixed population of PC (Fig 2B and [48, 55]) and to macrophage/microglia identified by *in situ* staining in infected mouse brain [45]. Because PC express EcoHIV for an extended period of time *in vivo* (Fig 2B), and persistently-infected macrophages are implicated in pathogenesis of several HIV morbidities in infected patients [39], we first clarified EcoHIV tropism to macrophages within mouse PC. To determine if EcoHIV tropism is determined solely by expression of its cellular receptor, we compared EcoHIV replication in mouse thymus and PC to that of ecotropic MLV, the donor of the gp80 envelope in EcoHIV (Fig 4A–4C). Thymus and PC express roughly similar amounts of CAT-1 RNA (Fig 4A); however, the two viruses replicate differently in these tissues. EcoHIV productively infects PC but not thymus; conversely MLV replicates extensively in thymus but poorly in PC (Fig 4B). To determine which cells among PC are susceptible to each virus, F4/80 bearing macrophages were purified for measurement of integrated EcoHIV and MLV DNA (Fig 4C and S2 Fig). EcoHIV infection is enriched in F4/80^+^ peritoneal macrophages (PM) compared to unseparated cells and MLV exclusively infects an F4/80-negative cell type within PC, potentially fibroblasts. Thus PC can serve as a surrogate for determination of EcoHIV infection in peritoneal macrophages. Evaluation of EcoHIV and MLV tropism in other mouse tissues is shown in S3 Fig and [68].

One route to facilitate profound SIV disease in macaques is antibody-mediated depletion of CD8^+^ T cells, potent antiviral effectors [69]. Under a similar strategy, we took advantage of nude (athymic) mice to investigate EcoHIV replication in macrophages when mature T cells, including CD8^+^ cells, are absent. Comparison of virus burden in PC in C57BL/6 and C57BL/6 nude mice in the first month of infection indicates that vRNA expression in PC declines in immunocompetent mice but increases when T lymphocytes are absent implicating T cell mediated control of EcoHIV expression (Fig 4D). More detailed analysis 3 months after EcoHIV infection shows that PC from nude mice had significantly higher burdens of both vDNA and vRNA including the spliced Vif transcript (Fig 4E). Using EcoHIV encoding enhanced green fluorescence protein, Fig 4F and 4G demonstrate that athymic mice also permit significantly higher viral protein expression in PM (P<0.05) than do immunocompetent mice. Confocal microscopy visualizes this viral expression in F4/80^+^ cells 25 days after infection (Fig 4G) These results indicate that macrophages are a prominent target for EcoHIV and that their spreading infection is partially limited by antiviral responses of T cells [45, 47].

### Macrophages carry inducible, replication competent EcoHIV

**Fig 5:**
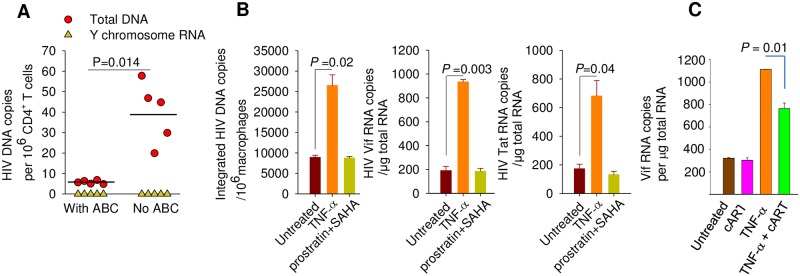
EcoHIV in macrophage reservoirs is transmissible *in vivo* and can be activated by TNF-α but not SAHA. **A.** Two weeks after transfer of PC from EcoHIV-infected male mice to females, females were euthanized and total vDNA and a Y chromosome transcript were measured in CD4^+^ spleen cells by QPCR. **B.** Eight weeks after EcoHIV infection, PM were isolated and stimulated by TNF-α or SAHA+prostratin for 48 h. Integrated vDNA and spliced *vif* and *tat* RNA were determined by nested QPCR and QPCR, respectively. **C.** Five weeks after EcoHIV infection PM were isolated and cultured either with no additions, 1 μM ABC and 1 μM RAL, TNF-α, or ABC, RAL, and TNF-α for 2 days prior to collection for QPCR detection of spliced *vif* RNA.

EcoHIV expression in PM (Fig 2) suggests that they are a source of infectious virus in chronically-infected animals. We addressed this possibility in two ways. We first inquired whether infected macrophages can transmit virus to CD4^+^ T cells *in vivo* (Fig 5A). We purified peritoneal macrophages from male nude/129X1 mice infected by EcoHIV and intravenously injected the cells to uninfected 129X1 females; a group of recipient mice was prophylactically treated with ABC to block new infection. Two weeks after adoptive transfer of macrophages, spleen CD4^+^ T cells were isolated from recipient mice and tested for EcoHIV vDNA burden, vRNA expression, and a Y chromosome transcript, the latter to detect any contaminating donor cells. The results show that EcoHIV was transmitted from infected macrophages to CD4^+^ T cells *in vivo* in a process requiring reverse transcription (Fig 5A), indicating that EcoHIV-infected macrophages can transmit virus *in vivo*; no donor cells were detected in the CD4^+^ T cell pool. Second, we tested whether virus in persistently infected macrophages can be further activated and produce infectious progeny virus. PC were isolated from mice 8 weeks after infection and cultured in the presence of either prostratin and SAHA, the most efficient inducers of latent EcoHIV in CD4^+^ cells (Fig 3), or TNF-α, an inflammatory cytokine that induces HIV expression in transformed human macrophages [70] (Fig 5B). As expected from the long-term kinetics of EcoHIV infection (Fig 2), PC continued to express vRNA without additional stimulation but prostratin and SAHA failed to further induce virus. However, TNF-α treatment increased the burden of both vDNA and RNA in PM, suggesting that further activation of transcription, presumably through NF-κB, induced progeny virus production and infection of new cells. To test the spread of infection into new cells, the experiment was modified to include culture of cells with ART; *vif* RNA was measured (Fig 5C). While ART has no effect upon the expression of vDNA in uninduced cells, it significantly reduced the amount of *vif* expressed after TNF-α induction indicating that progeny virus spread infection to new cells. These studies allow two conclusions to be drawn. Like CD4^+^ T cells, macrophages from infected mice produce infectious progeny EcoHIV. The EcoHIV reservoir in macrophages is active in transcription unlike the latent virus in CD4^+^ T cells.

### HIV infection of mice induces cognitive impairment which can be prevented but not reversed by ART

**Fig 6:**
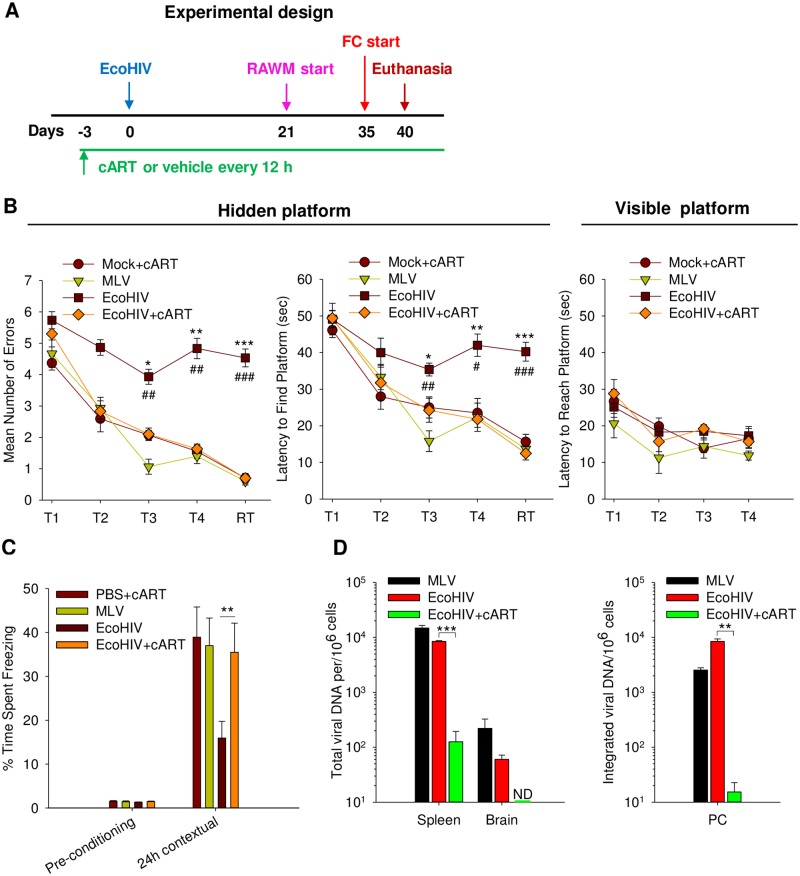
Both EcoHIV and MLV infect mouse brain but only EcoHIV-infected mice develop cognitive impairment. **A.** Design of the experiment. **B.** Groups of mice were gavaged with cART or vehicle before and during infection with indicated viruses or vehicle. At day 21 after infection, mice were tested for errors (left panel) and latency (middle panel) in finding the hidden platform, the right panel shows latency to reach the visible platform. RT represents the retention trial. **C.** Two days after completion of RAWM, the same mice were used to test contextual response to fear conditioning training. The percentage of freezing time was calculated for contextual fear memory deficit in each group of mice. **D.** Mice were euthanized on day 40 after infection and levels of total vDNA in spleen and brain measured by QPCR (left panel) and integrated viral DNA measured by nested QPCR in PM (right panel). All data are mean ± standard errors. *P<0.05, **P<0.01, ***P<0.001 EcoHIV vs. EcoHIV+cART; ^#^*P*<0.05, ^##^P<0.01, ^###^*P*<0.001 EcoHIV vs. MLV.

Persistently EcoHIV-infected immunocompetent mice have shown HIV DNA and mild pathogenic changes in the brain previously [48, 50, 51]. We now compared them to uninfected mice in behavioral tests designed to measure animal cognitive abilities widely used to model selected conserved human brain functions [71–74]. We used radial arm water maze (RAWM) designed to test working memory performance of infected mice during hippocampus-dependent visuospatial memory acquisition [75, 76] (reviewed in [77]), and contextual fear conditioning (FC) [78, 79] (reviewed in [80]) to assess amygdala system/hippocampus-dependent associative memory (Fig 6). The design of the experiment is shown schematically in Fig 6A. Four groups of 10 mice each were evaluated: EcoHIV-infected mice; MLV-infected mice serving as controls for the potential activity of gp80 envelope; mice treated with ART prior to and during EcoHIV infection, to test the requirement for viral replication in cognitive dysfunction; and uninfected mice treated with ART (Fig 6B and 6C). All four groups were first tested for their performance in RAWM starting 21 days after infection (Fig 6B). After the completion of RAWM testing, mice were allowed to rest for 2 days and then were subjected to FC tests (Fig 6C). EcoHIV and MLV infection was confirmed in spleen and brain after behavioral testing (Fig 6D). In the RAWM test, when assessed either by the number of errors made in finding the hidden platform (Fig 6B, left panel) or the time required to find it (Fig 6B, middle panel), only EcoHIV-infected mice showed statistically significant deficiency in spatial learning (T1-T4). The retention trials (RT) conducted 30 min after completing T4 trial indicate that the learning deficiency of EcoHIV-infected mice is due to defective working memory, which is required for learning a new hidden platform location on each subsequent day of testing [77]. MLV-infected and EcoHIV-infected+ART animals performed similarly in RAWM as mock infected+ART, indicating they maintained normal spatial learning capacity and had functioning working memory. All mice could readily find the visible platform indicating that all four groups were similar in their visual, motor, and motivational competence (Fig 6B, right panel). Tests of FC (Fig 6C), an independent cognitive test assessing fear neuronal circuit [73, 80, 81], also revealed defects only in EcoHIV-infected mice. Specifically, infected mice had reduced ability to remember the environment (the conditioning chamber) in which they had been subjected to fear stimulus the previous day, suggesting defective associative fear memory [80]. Systemic and brain viral burdens were similar in EcoHIV and MLV-infected mice; as expected ART almost completely prevented systemic EcoHIV replication and no virus DNA was present in the brain (Fig 6D). These findings indicate that EcoHIV infection causes impairment in the cognitive abilities tested, namely, visuospatial and working memory, and contextual fear memory, through HIV but not MLV-encoded functions. Because ART prophylaxis prevented cognitive impairments in EcoHIV-infected mice, our results also link EcoHIV replication in mice to development of cognitive disease.

**Fig 7:**
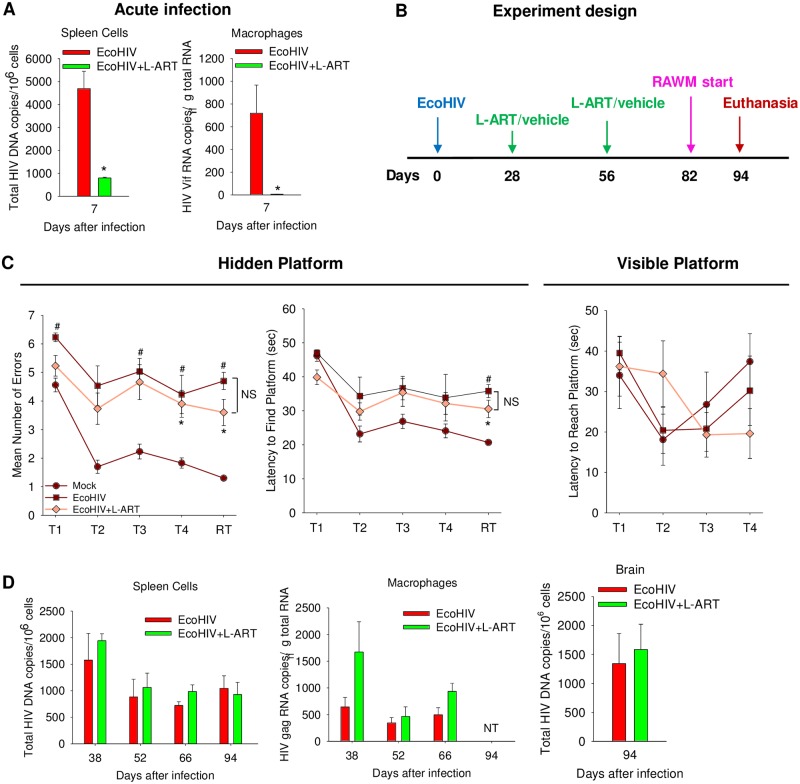
LASER ART (L-ART) fails to reverse HIV-NCI in chronically-infected mice. **A.** Two days prior to EcoHIV infection, mice were injected once with L-ART and their virus burdens in spleen and PC were evaluated seven days after infection. **B.** Design of the experiment. L-ART was administered twice, at 28 days and 56 days p.i. **C.** Results of RAWM conducted on chronically EcoHIV-infected L-ART-treated and untreated mice starting 82 days p.i.; L-ART treated, uninfected mice served as controls; left panel: errors; middle panel: latency; right panel: visible platform control. T represents the learning trial and RT the retention trial (*Mock vs. EcoHIV, *p*<0.002; #Mock vs. EcoHIV+L-ART, *p*<0.03). **D.** HIV burdens of treated and untreated EcoHIV-infected mice tested in spleen cells, peritoneal macrophages, and brain 94 days p.i.

To investigate whether NCI in persistently EcoHIV-infected immunocompetent mice resembles human NCI in display of ART-refractory phenotype, we tested the efficacy of long-acting combination-nanoparticles containing rilpivirine (RPV) and cabotegravir (CAB), called here long acting slow effective release ART (LASER ART, or L-ART), to affect HIV-NCI in infected mice (Fig 7). L-ART was administered at the dose previously shown to reduce plasma HIV loads by about 90% one month after treatment of HIV-infected humanized mice [82]. A single intramuscular injection of L-ART efficiently prevented acute EcoHIV infection *in vivo*, confirming its antiviral utility here (Fig 7A). We then tested the efficacy of L-ART to treat ongoing infection and NCI by injecting the drugs twice: 4 weeks and 8 weeks after EcoHIV infection; behavioral testing in RAWM was conducted starting 82 days p.i. (within the reported 30-day efficacy period of L-ART; [82]) and mice were euthanized 94 days p.i. (scheme in Fig 7B). Mice were bled to measure drug plasma concentrations (S2 Table) and groups of infected drug-free or L-ART-treated mice were euthanized at four times points to determine virus burdens (Fig 7D). L-ART was detectable at the time points tested in plasma as well as in brain tissue at euthanasia (S2 Table). EcoHIV DNA levels in spleen and vRNA levels in PC were similar to those illustrated in Fig 2; like the experiment shown in Fig 6, EcoHIV-infected mice manifested NCI, here assayed 3 months after infection and indicating lasting damage to brain function by this infection. However, despite its efficacy in prevention of EcoHIV infection, L-ART failed to affect virus burden in spleen or macrophages or NCI. The maintenance of EcoHIV NCI despite ART provides a platform in which to test interventions designed to ameliorate HIV-NCI that persists in infected individuals despite effective ART.

### In the absence of T cells, HIV-infected macrophages are sufficient for induction of cognitive impairment in mice

**Fig 8:**
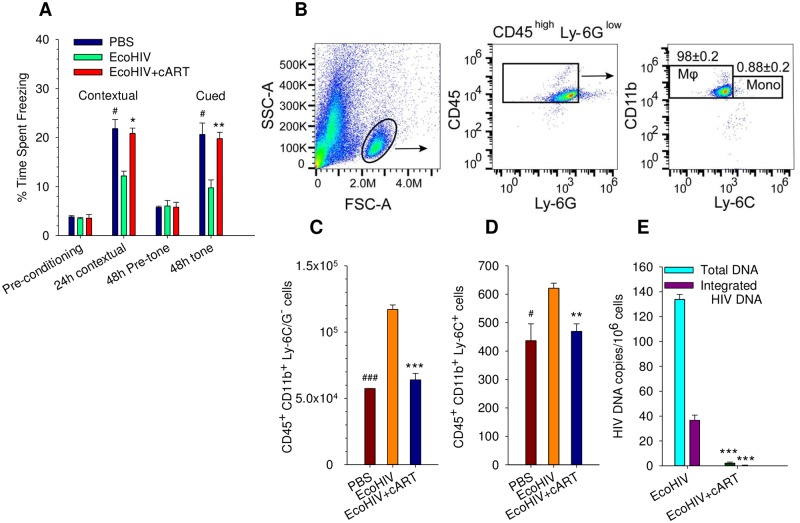
Infected mice lacking T cells develop cognitive impairment and have elevated number of monocytes/macrophages in the brain. **A.** Control and EcoHIV-infected nude mice with or without ART were tested 24 h after fear conditioning training for contextual fear response and 24 h later for cued fear response. *P<0.05, **P<0.01, EcoHIV vs. EcoHIV+ART; #P<0.05, EcoHIV vs. PBS. **B.-D.** Flow cytometry analysis of macrophage or monocytes in brains of nude mice with EcoHIV, EcoHIV with cART or PBS showing representative dot plots. Cell populations were gated based on isotype control antibodies. **B.** Staining for CD45 and CD11b and negative for Ly-6G/C. **C.** Staining for CD45, CD11b and Ly-6C and negative for Ly-6G. **D.** As determined by flow cytometry using 5 mice per group, the number of cells in each population per total mouse brain are shown. **E.** Total and integrated vDNA was measured by QPCR and nested QPCR, respectively, in infiltrating leukocytes isolated from the brain of EcoHIV-infected mice with and without cART. *P<0.05, **P<0.01, ***P<0.001.

CD8^+^ T cell depletion facilitates the development of encephalitis in SIV-infected macaques [83] and EcoHIV efficiently infects macrophages in mice lacking T cells (Fig 4) suggesting that these animals can also suffer HIV-associated NCI. Nude mice, treated with cART or untreated, were infected by EcoHIV and then were subjected to FC-associating the shocks either with conditioning surroundings for contextual fear memory (Fig 8A contextual panel) or with a tone for fear-tone associative memory (Fig 8A, 48 h tone panel); uninfected mice were tested in parallel. EcoHIV-infected nude mice suffered cognitive impairment as indicated by their poor ability to learn and recall associations, and this dysfunction required virus replication. Thus EcoHIV-infected nude mice, which display persistent productive infection in macrophages but have no T cells, suffer cognitive disease like infected-immunocompetent mice.

In macaques, the establishment of SIV infection in the brain correlates with the migration of monocytes from the periphery into the brain [84], consistent with generally accepted model of HIV neuroinvasion [67]. To investigate this mechanism in EcoHIV infection we isolated infiltrating leukocytes from the brains of EcoHIV-infected nude mice and determined their lineage by flow cytometry. The majority of cells expressed CD11b and CD45 but were low for Ly-6C and Ly-6G; indicating they are macrophages and not monocytes or neutrophils (Fig 8B). We addressed the extent of this brain infiltration by comparing the number of macrophages and monocytes in brains of uninfected, EcoHIV infected, and cART treated-infected mice (Fig 8C and 8D). EcoHIV-infected animals showed significantly more macrophages (CD11b^+^, CD45^+^, Ly-6C^-^, Ly^-^6G^-^) and monocytes (CD11b^+^, CD45^+^, Ly-6C^+^) infiltrating the brain than did either uninfected or infected ART-treated animals (Fig 8C and 8D, respectively). Infiltrating leukocytes were purified from brain tissue of the 3 mouse groups and tested for their total and integrated vDNA burdens (Fig 8E). It is clear that EcoHIV infects and integrates into leukocytes localized in the brain and that ART prevents this infection. These studies indicate that EcoHIV-infected nude mice suffer cognitive impairment despite their lack of T cells, that this infection increases macrophage/monocyte localization in the brain, and that these cell pools carry EcoHIV provirus. They strongly support the view that HIV infection of cells of the monocyte lineage drives HIV-associated brain disease [10, 39].

## Discussion

Our results demonstrate three major findings. First, EcoHIV establishes persistent infection in conventional mice, with virus present at low levels in CD4^+^ T cell and macrophage reservoirs and the brain. Second, infected mice manifest lasting HIV-specific cognitive impairment which can be prevented by antiretroviral drug prophylaxis, but which once established cannot be reversed by ART. Third, as shown by EcoHIV infection of mice lacking T cells, EcoHIV replication in macrophages *in vivo* is sufficient for the induction of cognitive impairment. We propose that EcoHIV infection of mice can serve as a small animal model of HIV-induced cognitive impairment in clinically asymptomatic human beings on successful ART [85] or in individuals with natural control of HIV replication [22] (Table 1).

**Table 1:** Features of EcoHIV-infected mice compared to HIV-infected people.

Infection feature	Persistently EcoHIV-infected mice	HIV-infected people on suppressive ART	HIV-infected people untreated
Viremia	No	No	Yes
Stable provirus in CD4^+^ T cells and macrophages	Yes	Yes	Yes
Provirus can be induced in CD4^+^ T cells by epigenetic modulators *ex vivo* and *in vivo*	Yes	Yes	NA
Persistent virus expression by infected cells	mØ only	No	Yes
Infectious virus can be rescued from T cells + mØ	Yes	Yes	Yes
Infectious virus can be transmitted from host to host	Yes	Yes/Low	Yes
Normal or improved CD4:CD8 ratio	Normal	Improved/Normal	No
At least partially functional immune system	Yes	Yes	No
Neurocognitive impairment (NCI)	Yes, 100%	Yes, 50%	Yes
NCI correlates with higher monocyte and macrophage levels in the brain	Yes	?	Yes

EcoHIV infection of mice reproduces some, but not all, aspects of HIV infection and disease in human beings and it resembles most closely infection of people on effective ART (Table 1). Notably, EcoHIV infection does not reproduce persistently high virus burdens in untreated people where patients with 200 CD4^+^ T cells per μl plasma carry roughly 2x10^5^ copies of viral DNA per 10^6^ tissue lymphocytes [86]. Without treatment HIV is robustly expressed by lymphocytes and macrophages in infected people [87]. Successful ART reduces virus burden to roughly 1,000 viral DNA copies per 10^6^ tissue lymphocytes in people [86] most of which is latent [88]. In comparison, untreated EcoHIV-infected mice show an initial burst of HIV expression by lymphocytes followed by a decline to a persistent burden of about 1,000 viral DNA copies per 10^6^ tissue lymphocytes most of which is integrated and latent. Like people on effective ART, chronically-infected mice have normal CD4^+^ T cell levels and are immunocompetent but in mice this is independent of ART. A striking characteristic of EcoHIV infection is persistent virus expression by macrophages (Figs 2 and 5). The basis for the discrepancy between EcoHIV status in lymphocytes and macrophages is unknown but is potentially critical to the observations here. Thus of two elements that largely govern HIV disease, CD4^+^ cell depletion leading to immunodeficiency and viral expression and response by macrophages leading to brain disease, only the latter is well preserved in EcoHIV infection of mice. Replication in macrophages and nervous system disease are characteristic of other lentiviruses [89].

Previous studies in culture and in transgenic rodents showed blocks to HIV replication at the level of DNA nuclear localization, integration, transcription, RNA transport, and protein processing [90–92]. Among these restrictions is a replacement in Cyclin T1 of an essential cysteine found in humans by tyrosine in mice and rats that prevents efficient Tat binding and initiation of TAR recognition and transcription [93]. However, rat cells show highly efficient Tat-mediated transcription [94] and rat macrophages are susceptible to productive HIV infection comparable to human cells *in vitro* through VSV pseudotypes [94] and *in vivo* in CD4^+^/CCR5 transgenic animals [95]. The results presented here clearly show that EcoHIV completes its life cycle in infected murine CD4^+^ T cells and macrophages *in vivo* producing the major DNA forms, spliced and unspliced RNA, viral protein, and *in vivo* transmissible progeny virus (Figs 2–6); the virus persists in infected cells essentially for the lifetime of the mouse host. The frequency of virus expressing T cells declines sharply within weeks of infection while anti-HIV antibodies and T cells are induced (Fig 2). These T cell responses tend to reduce virus expression as shown in the increased virus burden in PC observed in athymic mice (Fig 4) and as also shown in the demonstration of virus-suppressive CD8^+^ cells 6 weeks after infection [47]. However, the inefficient infection of CD4^+^ T cells and the absence of significant viremia during the early peak of EcoHIV infection (Fig 2) indicate additional constraints of HIV infection of mice. A restriction that is seen in EcoHIV infection and requires investigation is poor virion export. It may be due to a block to budding by murine tetherin since unlike human tetherin, it is poorly antagonized by HIV Vpu [96]. Overall findings from other laboratories and those presented here indicate that HIV restriction in rodents is not an all or nothing phenomenon.

It is noteworthy that EcoHIV tissue tropism in mice is not controlled solely by cellular display of CAT-1, the receptor for MLV and EcoHIV. MLV and EcoHIV replicate in different cell types, despite their common envelope. Within PC, EcoHIV infects F4/80^+^ macrophages while MLV exclusively replicates in the F4/80 negative pool. Within other tissues, EcoHIV replication is observed only where lymphocytes or macrophages are found. These observations indicate that EcoHIV tropism in mice resembles that of HIV in humans, at least with respect to its ability to replicate in principal host cells implicated in HIV pathogenesis, namely, CD4^+^ T cells, macrophages, and as shown elsewhere [45] myeloid cells in the brain. Murine retrovirus tropism is conferred by both envelope and the LTR [97] and there is recent evidence that LTR sequences distinguish R5 and X4 HIV species [98], suggesting that LTR activity and use of host cell transcription factors contribute to EcoHIV tropism in mice.

Despite impaired infection, murine T cells (Fig 3) resemble infected human T cells [26] in their ability to silence proviral transcription *in vivo* and both cell populations can be induced to virus expression *ex vivo* by a combination of mitogens, prostratin, and chromatin remodeling agents VPA or SAHA (Fig 3 and [62, 65]), suggesting that in these cells proviral DNA locates in inactive chromatin regions. Treatment of EcoHIV-infected mice with activating antibodies and prostratin and either SAHA or valproic acid induced viral transcription and protein expression in T cells (Fig 3F); notably, similar activation using the histone deacetylase inhibitor parobinostat in humanized mice did not activate virus expression [99]. In mice, macrophages support EcoHIV transcription longer than do T cells. TNF-α further activates virus expression in macrophages, while prostratin/SAHA did not indicating that EcoHIV integrates into transcriptionally active chromatin in macrophages (Fig 5). Persistence as silent provirus in T cells and maintenance of vRNA production in macrophages during effective antiviral immune responses suggest that these responses induced within weeks of infection promote the development of viral reservoirs and, as discussed below, virus within these reservoirs is refractory to ART. EcoHIV infection of mice may provide an additional platform for investigation of means to eliminate latent HIV in T cells either by the “shock and kill” approach [100, 101] or by excising provirus [102, 103] as well as for further study of the active reservoir of HIV seen in macrophages here and in other animal model systems [82, 104, 105].

To directly determine the production of infectious virus by EcoHIV-infected mouse cells, either macrophages or CD4^+^ cells were adoptively transferred from infected males to uninfected female mice. Recipients were productively infected in cell populations different from those transferred and the absence of an abundant (1-5x10^5^ copies per μg RNA) Y chromosome transcript in infected recipient cells excludes contamination by donor cells. Analogous results were obtained in culture employing ART to prevent virus spread from lymphocyte cocultivation or from TNF-α-induced macrophages (Figs 3B and 5C). These findings demonstrate that progeny virus produced by cells infected *in vivo* is infectious as also was demonstrated in EcoHIV transmission by mouse mating [54].

Some investigators suggested that the observed infection of macrophages by HIV in humans and animals is a result of phagocytosis of infected T cells and not direct infection [106]. The highly efficient replication of EcoHIV in CD11b^+^ macrophages in nude mice reported here makes it clear that HIV, like other lentiviruses [107], targets these cells in infected hosts. Our findings echo studies of HIV replication in myeloid-only humanized mice [108] and quantitative infectious SIV recovery from various macrophage populations in infected macaques [105]. Not only is there convincing evidence that macrophages are susceptible to HIV/SIV, but a recent report indicates that their infection may survive ART. In human beings, HIV was frequently found to establish infection in macrophage-rich tissues, including 48/87 brain tissues examined at autopsy, despite ART driving viremia below detection [109]. These findings underscore the necessity to control virus expression by macrophages in the goal to control HIV pathogenesis in people living with HIV. In this regard, study of EcoHIV infection of mice may be useful.

The present work continues identification of the cognitive, virological, and mouse host parameters of EcoHIV-infected mice. First considering cognitive deficits, here termed murine HIV-NCI, EcoHIV-infected mice failed to perform in two functionally independent tests of cognitive ability, FC test of associative memory [73, 80] and RAWM task of visuospatial memory [75, 77] recently employed also in evaluation of 6-diazo-5-oxo-L-norleucine prophylaxis of murine HIV-NCI [110]. The impaired ability of infected mice to complete the delay/retention trials in the spatial environment of RAWM as changed daily (Fig 6) also suggested dysfunction of working memory during spatial learning [77]. Spatial learning, spatial memory, and working memory are three of seven cognitive ability criteria evaluated during neuropsychological (NP) diagnosis of mild NCI [6] and they are frequently affected in patients on HIV-suppressive ART (reviewed in [9, 10]). The impairment of associative memory in infected mice shown in FC behavioral tests may be indicative of neuropsychiatric disorders also affecting HIV-positive individuals [111–114]. Although rodents and rodent behavioral tests do not fully reproduce the complexity of human behavior, our results indicate that murine HIV-NCI reflects some cognitive abnormalities seen in HIV-positive patients. It is noteworthy in this context that a virtual reality-based water maze test was more sensitive than standard NP evaluation in detecting early spatial memory deficits in HIV-positive women [115], indicating that HIV infection imposes similar dysfunction in humans and rodents in the conserved ability domain of spatial navigation [116] and implying that they use similar complex behavioral strategies for solving spatial problems [117]. Further validation of murine HIV-NCI as a model of human NCI will require comparative analysis of affected brain regions and neuronal networks involved in the cognitive deficits observed.

Addressing the virological aspects of murine HIV-NCI, both EcoHIV infection and disease were prevented by ART (Fig 6), demonstrating the requirement for HIV replication *in vivo* for disease development and, conversely, excluding a passive role of input virus in this pathogenesis. The intact cognitive function of MLV-infected mice indicated that HIV functions alone (and not MLV gp80 expressed by EcoHIV) are responsible for murine HIV-NCI; and further that gp120 absent from EcoHIV is not required. Tat is expressed by EcoHIV-infected mice [48] and based on its ability to cause cognitive dysfunction in transgenic mice [118, 119] may contribute to murine HIV-NCI observed here.

To our knowledge, induction of NCI by EcoHIV infection of conventional mice is the first experimental animal system that may allow modeling host pathophysiological conditions for mild NCI induction in HIV-suppressed people. The fundamental finding of our work is that EcoHIV infection causes NCI but not CD4^+^ T cell depletion or immunodeficiency in mice (Fig 2), separating at least part of the causes of these two HIV disease manifestations. In this respect, murine HIV-NCI resembles HIV-NCI observed one year after HIV infection in about 20% of ART-naive people with normal CD4^+^ T cell counts [20–22]. The frequency of NCI in such subjects who began ART one year after infection was similar to that in subjects who delayed treatment until their CD4^+^ counts declined to a predetermined low threshold [23], suggesting that mild HIV-NCI develops both independently of immunodeficiency and despite ART. Similarly, mild cognitive disease has been observed in some HIV elite controllers, who naturally control HIV replication and maintain immune-sufficiency [120]. As shown here, murine HIV-NCI also develops regardless of immune competence and ART. ART begun 4 weeks after EcoHIV infection of mice did not affect either virus burden or the extent of loss of spatial learning and memory seen in RAWM 2 months later (Fig 7). The inability of ART to reduce EcoHIV burdens during 2 months of treatment likely reflects the residence of virus in reservoirs insensitive to reverse transcriptase or integrase inhibition (Figs 3 and 4). We propose that EcoHIV infection of conventional mice, with or without ART, reproduces at least some of the conditions for the HIV-NCI that develops in 50% of people despite ART and restored immunity (Table 1).

Finally, the ability to experimentally infect mice with EcoHIV and induce HIV-NCI allowed us to delineate some of the roles of cells of the monocyte lineage in the development of the disease. Infected macrophage/monocytes have long been suspected of transporting HIV into the brain [67] and the dynamics of their infection, subsets of cells involved, chemokines and cytokines produced, and changes in brain anatomy are being elucidated by many investigators [28]. The development of cognitive deficits in EcoHIV-infected athymic mice shown here indicates that infection of the macrophage lineage, independently of infection of T cells, is sufficient to cause brain disease. We also showed that the number of macrophages migrating to the brain increases within weeks of systemic EcoHIV infection. Previous neuropathological studies of brain sections and cellular phenotypes from HIV-infected people have reached similar conclusions [121], however we isolated infiltrating cells from the entire brain to count and identify them as well as measure their virus burden. This animal model can provide a versatile tool to evaluate interventions to block macrophage migration and establishment of HIV infection in the brain and neuropathogenesis.

## Materials and methods

### Ethics statement

After obtaining written informed consent from all subjects, peripheral blood was obtained from the study participants in accordance with AIDS Clinical Trials Group (ACTG) protocols which was reviewed and approved by an Institutional Review Board of St. Luke’s-Roosevelt Hospital Center. All human subjects in the study were adult. All animal studies were conducted with the approval of the St. Luke’s-Roosevelt and Mount Sinai School of Medicine Institutional Animal Care and Use Committee (IACUC), protocol IACUC-2014-0124, and in full compliance with NIH guidelines.

### Human PBMC

Peripheral blood mononuclear cell (PBMC) were isolated from fresh heparinized blood by Ficoll-paque plus (GE Healthcare, Pittsburgh, PA) gradient centrifugation. Purified PBMC were used for both DNA and RNA analysis.

### Antiviral drugs and inducers

ABC (LGM Pharma, Nashville, TN) was purchased as a solid and diluted in water. RAL (Isentress tablets; Merck Sharp & Dohme Corp., Whitehouse Station, NJ) was purchased as 400 mg tablets, dispersed and diluted in water. As indicated RAL was used at 2.5 mg, 10 mg, or 100 mg/kg/day in mice, ABC was used at 150 mg/kg/day in mice by gavage (Fig 1C–1F) or at 150 μM *ex vivo* culture; for treatment of macrophages *ex vivo* ABC and RAL were each used at 1 μM. Nanoparticles containing cabotegravir and rilpivirine, here termed long acting slow effective release ART (LASER ART, abbreviated L-ART) were prepared as described in detail [82,122] and administered at 45 mg/kg for each drug by intramuscular injection either 2 days prior to infection for prevention or 4 and 8 weeks after infection. Plasma was collected as indicated and drug concentrations were determined by ultra-performance liquid chromatography tandem mass spectrometry (S2 Table)[122]. For inducers (Selleckchem, Houston, TX), 5’-azacytidine, valproic acid and vorinostat were suspended in sterile water and prostratin was dissolved in DMSO and diluted in water.

### Mice, infection and treatment

Adult male and female C57BL/6J, 129X1/SvJ and nude mice (Foxn1nu/Foxn1nu), all 8–12 weeks old, were purchased from The Jackson Laboratory (Farmington, CT). Systemic infection of mice was conducted by intraperitoneal injection of virus stock and samples were harvested as described [48]. For antiretroviral therapy with free drugs, ART suspensions prepared as described above were administrated by oral gavage.

For *in vivo* reactivation of latent EcoHIV, a dose of 1.2 mg/kg of anti-CD3/28 or the isotype mAbs was injected subcutaneously every 2 days for each mice, and 0.8 mg/kg of prostratin and 48 mg/kg of SAHA was administrated by intraperitoneally and gavage, respectively, once per day.

### Plasmids, virus, and infection

All the three plasmids used in our study including EcoHIV/NDK [48], EcoHIV/NL4-3-GFP [45] and pNCS (Mo-MuLV) [123] were described previously, and referred to as EcoHIV, EcoHIV-GFP and MLV, respectively (S5A, S5B, S5E Fig). The full nucleotide sequence of EcoHIV/NDK (EcoNDK) was submitted to GenBank (NCBI-NIH), accession number MG470653.1. EcoHIV-Luc (S5C Fig) was constructed by introducing a luciferase gene (GB: AAA89084, from the vector pGL3 Basic) and an internal ribosomal entry site (IRES) into the Nef locus of EcoHIV/NDK backbone. The EcoHIV-ΔENV (S5D Fig) was constructed by introducing two stop codons followed ATG of the coding region of signal peptide based on the EcoHIV/NDK. The resulting plasmid was cotransfected with plasmid pVSV-G at a 1:1 ratio (wt/wt) to generate pseudotyped virus capable of a single-round infection. All virus stocks were prepared by transfection of plasmid DNA into 293T cells and titered for p24 core antigen content as described [48]. To obtain similar infectivity comparable with EcoHIV in mice, MLV stocks were titered on rat XC cells and normalized to EcoHIV stocks by ENV RNA copies from RT-QPCR [124]. EcoHIV was inactivated by heating at 57°C for 45 min. All viruses were inoculated intraperitoneally.

### Cell isolation, culture, and treatment

Mice were euthanized with carbon dioxide and spleens as well as peritoneal cells (PC) were collected from mock- or EcoHIV-infected mice. Then, spleens were homogenized into single-cell suspensions by autoclaved frosted glass slides in cold RPMI medium, which were filtered through a nylon cell strainer (70-μm diameter, Becton Dickinson, Franklin Lakes, NJ). Both of the cell pellets were washed once with PBS and erythrocytes were lysed for 3 min in ACK lysing buffer (BioWhittaker, Walkersville, MD). CD4^+^ T lymphocytes were enriched by negative depletion (Dynal Mouse CD4 Negative Isolation Kit, ThermoFisher Scientific, Waltham, MA). Resting CD4^+^ T lymphocytes were further isolated from total CD4^+^ T cells by negative selection using anti-CD69, anti-CD25 and anti-MHC class II (I-A) antibodies from Southern Biotech (Birmingham, AL) to remove the activated cells. Monocytes were purified by negative selection using the EasySep mouse monocyte enrichment kit (Cat #: 19861, StemCell Technologies, Cambridge, MA), according to the manufacturer’s instructions. Peritoneal macrophages were plated on petri dishes with DMEM supplemented 20 ng/ml recombinant mouse M-CSF (Biolegend, San Diego, CA) and cultured for 6 h. After removal of non-adherent cells by washing, the macrophages were harvested. Purity of CD4^+^ T cells and peritoneal macrophages were verified by flow cytometry and was typically greater than 94% and 98%, respectively (S2A and S2B Fig).

Brain-infiltrating mononuclear cells were isolated as described previously [125]. Briefly, animals were perfused with 30 ml of ice-cold PBS, and brains were collected. A single-cell suspension was obtained by grinding the tissues using 1 ml syringe plunger and passed through a 70-μm strainer. Mononuclear cells were obtained by Percoll gradient (30/70%, Sigma, St. Louis, USA) centrifugation at 500 g for 30 min at room temperature (20–25°C) collected from the interphase, washed and used for further analysis.

For *ex vivo* virus reactivation, the resting CD4^+^ T cells were first stimulated using Dynabeads Mouse T-activator CD3/CD28 (ThermoFisher Scientific) for 2 days and then treated for 2 days with the following concentration of inducers: 5 μM 5’-azacytidine, 1 μM valproic acid, 1 μM SAHA, 1 μM prostratin and 100 ng/ml TNF-α. For cytokine stimulation of macrophages, we cultured purified cells with 100 ng/ml TNF-α.

### Nucleic acid purification

Spleen, cell, macrophage, and viral DNA was isolated using DNAzol according to the manufacturer’s instructions (ThermoFisher Scientific). Brain DNA was isolated with modifications: one hemisphere was homogenized in 0.4 ml Trizol (ThermoFisher Scientific). To 0.1 ml homogenate, 0.9 ml DNAzol was added, mixed, and then 0.5 ml 100% ethanol was added to precipitate DNA. DNA was pelleted by centrifugation at 140,000 rpm for 15 min, washed twice in 1.5 ml 75% ethanol and resuspended in 100 μl 8 mM NaOH. After resuspension, the lysates were neutralized by the addition of 10 μl 0.1M HEPES to pH 8.0. Total cellular RNA was purified using an RNeasy mini kit and total RNA from animal tissues was purified using the RNeasy lipid tissue mini kit according to the manufacturer’s protocol (Qiagen, Valencia, CA).

### Quantification of HIV total DNA and 2-LTR circular DNA

QPCR quantitation of total HIV DNA in EcoHIV/NDK-infected mice and DNA standardization was described [55]; HIV 2-LTR circular DNA was measured by QPCR as described [45]. The measurement of HIV-1 total DNA in human PBMC has been previously described [56] and the quantification for total MLV DNA was conducted as described [124]. DNA from human cells was standardized by QPCR amplification of human β-globin QPCR (ThermoFisher Scientific) and custom-designed mouse β-globin QPCR [48].

### Quantification of integrated HIV-1 DNA in mouse and human genome

The analysis of HIV-1 integration level in human cells was reported in detail previously [56]. To quantify integrated EcoHIV in mouse cells, we (i) prepared the integration standard curve and used two-step PCR amplification including first (ii) a 12-cycle nonkinetic preamplification followed by (iii) nested real-time PCR assay. The procedure is depicted schematically in Fig 1A. (i) Preparation of the integration standard (IS) DNA. Mouse RAW 264 cell line was infected with high-titer stock of VSV-G pseudotyped EcoHIV-ΔENV virus and cultured *in vitro* for 30 days to ensure degradation of extrachromosomal forms of viral DNA. The number of integrated EcoHIV DNA copies per cells was then determined by quantifying the amount of total HIV-1 DNA by real-time PCR [55] and it was found to be 1 copy/cell. The IS DNA was then diluted with spleen cell DNA from uninfected mice to keep the number of *B1* sites per reaction constant. (ii) 12-cycle non-kinetic preamplification. In this step, the IS and the unknown samples were amplified for 12 cycles. In addition, for each unknown sample, a control reaction in which the mouse repeat element [57] primer pair (B1-F and B1-R) was omitted was run in parallel. Standard curves were prepared by making serial 5-log dilutions of IS DNA. The preamplification primers were: fNef, 5’-TACAAAGAAGCTGTTGATCTTAGCC-3’; murine cellular anchor primer B1-R, 5’-CCGAGTGCTGGGATTAAAGG-3’; and B1-F, 5’-CCAGGGCTACAGAGAAACCCTGTC-3’. The conditions for first-round PCR reaction were: a 2-min hot start at 94°C followed by 12 steps of the following: denaturation at 94°C for 30s, annealing at 50°C for 30s, and extension at 68°C for 16 min. (iii) Nested real-time PCR. The 2 μl preamplification product including both IS standard and unknown samples was supplemented with the following primers and a probe: LTRF, 5’-CTGGCTAATTAGGGAACCCACTG-3’; LTRR, 5’-GGACTAAACGGATCTGAGGGATCTC-3’; and the LTRP, 5’-(FAM)-TTACCAGAGTCACACAACAGACGGGCA-(MGBNFQ)-3’. The thermal program was 2 min at 50°C, 10 min at 95°C, and 40 cycles of 15 s at 95°C and 1 min at 60°C. Data analysis was performed with the 7500 System Software (ThermoFisher Scientific). The number of proviral copies in the unknown sample is the number of the total number of copies minus the number of copies in the control reaction. All samples were run in duplicate. HIV integrants were normalized to the number of cells in the sample determined in a parallel amplification of murine β-globin DNA.

### EcoHIV transmission by adoptive cell transfer

To determine whether macrophages and CD4^+^ T cells harbored replication competent EcoHIV *in vivo*, peritoneal macrophages and spleens were harvested from EcoHIV-infected male 129X1 mice 3 weeks after infection and the purified CD4^+^ T cells and macrophages were injected into tail veins of nude female and 129X1 female mice, respectively, which were prophylactically treated with ABC or water by gavage. Two weeks after cell transfer, peritoneal macrophages were isolated from female nude mice for measurement of EcoHIV *gag* RNA or a ubiquitous Y chromosome transcript as described [54]. Meanwhile, the CD4^+^ T were purified from spleen of 129X1 female mice. The cell-associated EcoHIV *gag* DNA and Y chromosome transcript was quantified.

### Viral outgrowth assay

To determine whether CD4^+^ T cells harbored replication competent EcoHIV *ex vivo*, a viral coculture assay was designed Briefly, CD4^+^ T cells were isolated from EcoHIV-infected mice at day 25 after infection and were activated with anti-CD3/CD28 beads (ThermoFisher Scientific) for 2 days. CD8-depleted splenocytes from uninfected mice were stimulated with 1 μg/ml Concanavalin A (Sigma) and 1 ng/ml IL-2 (Roche Applied Science, Indianapolis, IN) for 2 days. We cultured 1x10^6^ purified CD4^+^ T cells with 1x10^6^ CD8^+^-depleted splenocytes in the presence of 150 μM abacavir for 3 days. The cell-associated EcoHIV DNA was measured by QPCR.

### Detection of anti-HIV/NDK immune responses by Elisa or by cytokine induction of T cells

Anti-HIV/NDK Gag IgG antibodies in mouse sera were detected by Elisa as described [54,124]. All sera were tested at 1:20 dilutions and the data reported represent OD readings with the reading obtained in individual pre-bleeds subtracted. Spleen cells from infected or mock infected mice harvested at the time indicated in the text and then were stimulated with clade D HIV Gag peptides in culture and then subjected to flow cytometry measuring intracellular interferon-γ and surface expression of CD4^+^ and CD8^+^ as described [46].

### Flow cytometry

For surface staining, samples were washed in staining buffer (PBS containing 1% BSA, 5% mouse serum, 0.1% sodium azide), followed by incubation with anti-Fc receptor (BD Biosciences clone 2.4G2, 1 μg/ml), and then stained for 30 min on ice in the dark with 50 μl of an antibody cocktail. The following fluorochrome-conjugated antibodies were used: anti-mouse CD3-RPE-CY5 (ThermoFisher Scientific clone 145-2C11, 1:100), anti-mouse CD4-FITC (ThermoFisher Scientific clone GK1.5, 1:6), anti-mouse F4/80-PE (Miltenyi Biotec clone REA126, 1:6), anti-mouse CD11b-APC (Miltenyi Biotec clone M1/70.15.11.5, 1:6), anti-mouse CD25-PE (ThermoFisher Scientific clone 7D4, 1:6), anti-mouse CD69-APC (ThermoFisher Scientific clone H1.2F3, 1:6), anti-mouse CD45-FITC (ThermoFisher Scientific clone 30-F11, 1:100) and anti-mouse Ly-6C-PE (ThermoFisher Scientific clone HK 1.4, 1:40). The autofluorescence and isotype controls was performed in parallel. After 2 washes, the cells were fixed in 1.2% paraformaldehyde for 2 h at 4°C. Data were collected by BD Accuri C6 Flow Cytometer and analyzed with FlowJo (Tree star, Ashland OR).

### Immunofluorescence microscopy

Peritoneal macrophages were plated and cultured on chamber slides with DMEM plus 20 ng/ml recombinant mouse M-CSF for 6 h. After washing 3 times with PBS, the slides were fixed for 30 min with 3% paraformaldehyde in PBS and microscopy for detection of EcoHIV-GFP was conducted as described [45].

### Radial-arm water maze (RAWM) testing

Behavioral studies to assess spatial learning and memory were performed in a six-arm radial arm water maze using a standard protocol including a platform submerged beneath opaque water and a set of visual cues at the end of each maze arm [75, 126, 127]. RAWM tests subject’s learning/memory in a complex spatial environment in which both the location of the hidden platform and the lane where mice are first placed are randomly changed on each day of testing [75, 77]. This compels the animals to use working memory to learn a new platform location each day during the four training (T) trials (rather than using long term reference memory of fixed platform location as in a standard water maze); the proficiency in working memory at the end of the testing day (that is, the ability to remember the platform location as learned on a given day) is then directly assessed in the 30 min-delayed retention trial that follows training trials [75, 77].

Each testing group contained 10 mice; and RAWM testing consisted of 4 training trials (T) of 60 s and 1 post-training 60 s retention trial (R) administered after 30 min rest. The hidden platform was rotated randomly to a different arm each test day to ensure that mice used working memory to locate the platform. Testing was considered complete when control mice reached asymptotic performance of one error or fewer in finding the hidden platform on trials T4 and R (9–10 days for 129X1 mice). Errors for the last three days of testing were averaged and used for statistical analysis. Each of the training trials began by placing a mouse randomly into one of the 6 swimming arms and allowing the mouse to swim for 60 s to find the hidden platform, during which time the number of errors (entering an arm without the platform and/or 20 s of immobility) and latency to locate the hidden platform were recorded. The retention test was performed in the same manner as the learning trials. The hidden platform tests were followed by measuring the time (latency) it took for treated and control mice to find a visible platform, as a control for possible effects of treatment on animal vision, motivation, or ability to swim to the platform.

### Contextual and cued fear conditioning assessment

As times after infection indicated in the text, mice were tested in a contextual and cued fear conditioning paradigm as described previously[73, 80]. Briefly, an individual mouse was placed in the center of freeze monitor (35.5x38.1x31.75 cm) and allowed to explore freely for 3 min, during which the computer program was initialed. Fear conditioning was conducted with four (three for 129X1/SvJ) pairing of a 30 s, 80 dB auditory cue co-terminating with a 2 s, 0.5mA scrambled foot shock (US). The inter-trial interval (ITI) was 70 s. Two min after conditioning, mice were returned to the home cages. After each test, freeze monitor was cleaned with 70% ethanol to reduce residual stress odors. Contextual testing was conducted 24 h after conditioning in the same chamber. Cued fear conditioning was conducted 48 h after training in a novel black triangular chamber sprayed with 10% imitation vanilla flavored extract (McCormick & Company, Inc., Sparks, MD) and wiped down between trials. The time of the first three photobeam breaks was recorded by the SDI software (San Diego Instruments, Inc., San Diego, CA) and the percentage of time spent freezing (not breaking photobeams) was calculated over the 5-min period in contextual conditioning and during the pre-tone and tone test (three trials, 10 s/trial, 70 s ITI) in cued conditioning for analyzing freezing behavior.

### Statistical analysis

Data were analyzed using Excel and Student’s paired t test. A P value of <0.05 was considered significant, and all results are presented as means ± standard errors of the means (SEM). *p<0.05, **p<0.01, ***p<0.001.

## Supporting information

S1 Fig Anti-FITC responses are similar in EcoHIV-infected and control mice. Four mice each were injected with EcoHIV or PBS; one month later mice were immunized by IP injection with *E*. *coli* cells labeled with FITC. Mice were bled from the retro-orbital sinus before EcoHIV or PBS injection and at 10 and 17 days after immunization. Anti-FITC antibody response in 1:20-fold diluted sera was measured by Elisa with plates coated with ovalbumin FITC. Data presented are net OD after subtraction of the prebleed values, each point represents one mouse.
(PPTX)

S2 Fig Assessment of the purity of isolated mouse cells used in this work. (A). CD4^+^ T cell population were negatively isolated from splenocytes and analyzed by flow cytometer. (B). Peritoneal cells were isolated and cultured in DMEM with 20 ng/ml M-CSF for 6 h and the adherent cells were harvested by Cellstripper and analyzed by flow cytometer. Numbers indicate the percentage of gated cells. (C) Resting CD4^+^ T lymphocytes were further isolated from total CD4^+^ T cells and identified by flow cytometer using anti-CD69, anti-CD25.
(PPTX)

S3 Fig EcoHIV integration frequency in mice resembles that of HIV in PBMC of patients on long-term ART. **A.** In panels left to right total HIV DNA was measured by QPCR, integrated DNA was measured with nested QPCR, and genomic vRNA was measured by QPCR in PBMC from HIV patients with average CD4^+^ T cell counts more than 500/μl blood. The line represents the mean value. **B.** The ratio of integrated to total vDNA for each patient sample or each mouse sample more than 2 months after infection (Fig 2A) and then the mean ratios of groups were obtained. **C.D.** At 6 weeks after EcoHIV infection, mice were treated with vehicle or abacavir and raltegravir for 14 days prior to tissue collection. Integrated EcoHIV DNA was measured in spleen (**C.**) or PC (**D.**). The horizontal bar represents the median of these values.
(PPTX)

S4 Fig Despite sharing gp80 envelope with MLV, EcoHIV maintains HIV tropism. (**A-D**)**.** Ten days after EcoHIV or MLV infection of mice, the indicated tissues were harvested for measurement of viral nucleic acids by QPCR. (**A**) 2LTR circular DNA, (**B**) integrated viral DNA, (**D**) ENV RNA and (**E**) Spliced *vif* RNA. (**E**) At 7 d after EcoHIV-EGFP or MLV-EGFP infection of mice, peritoneal cells were analyzed for F4/80 and intracellular EGFP expression. Numbers in the flow plots indicates the percentage of gated cells expressing EGFP. Red histograms are isotype controls. BM = bone marrow, SP = spleen, PC = peritoneal cells, LN = lymph nodes, TH = thymus; LI = liver, LU = lung.
(PPTX)

S5 Fig EcoHIV and MLV genomes. (A-D). The genomes of EcoHIV/NDK (A) and variants in (B), (C) and (D) are derived from the molecular clone HIV-1/NDK [127], in which the HIV genes are shown in blue and the MLV gp80 was shown in red and black (deletion). The internal ribosome entry site (IRES) shown in (B) and (C) permits expression of EGFP and luciferase from the HIV RNA transcript. (D) Was constructed by introducing two stop codes followed ATG of the coding region of signal peptide based on (A). E-G. The genomes of MLV variants in (G) was derived from (F) by introducing two stop codes followed ATG of the coding region of signal peptide in gp80. 2A peptide in (F) and (G) was derived from porcine teschovirus-1.
(PPTX)

S1 Table Clinical profiles of the HIV-1-infected patients on suppressive ART. (PPTX)

S2 Table L-ART plasma and brain tissue concentrations in EcoHIV-infected mice. For L-ART pharmacokinetics, plasma samples and brain tissues were collected as indicated and tested by ultra-performance liquid chromatography tandem mass spectrometry for drug concentrations. The samples were from experiment depicted in Fig 8; 3–4 mice were sampled per collection time.
(PPTX)
