# Supplementary material for: EcoHIV infection of mice establishes latent viral reservoirs in T cells and active viral reservoirs in macrophages that are sufficient for induction of neurocognitive impairment
Source: PLoS Pathog. 2018 Jun 7;14(6):e1007061. doi: 10.1371/journal.ppat.1007061 (PMC5991655; doi:10.1371/journal.ppat.1007061)
Supplement: S1 Fig — Four mice each were injected with EcoHIV or PBS; one month later mice were immunized by IP injection with E. coli cells labeled with FITC. Mice were bled from the retro-orbital sinus before EcoHIV or PBS injection and at 10 and 17 days after immunization. Anti-FITC antibody response in 1:20-fold diluted sera was measured by Elisa with plates coated with ovalbumin FITC. Data presented are net OD after subtraction of the prebleed values, each point represents one mouse. (PPTX) [file ppat.1007061.s001.pptx]

## Slide 1
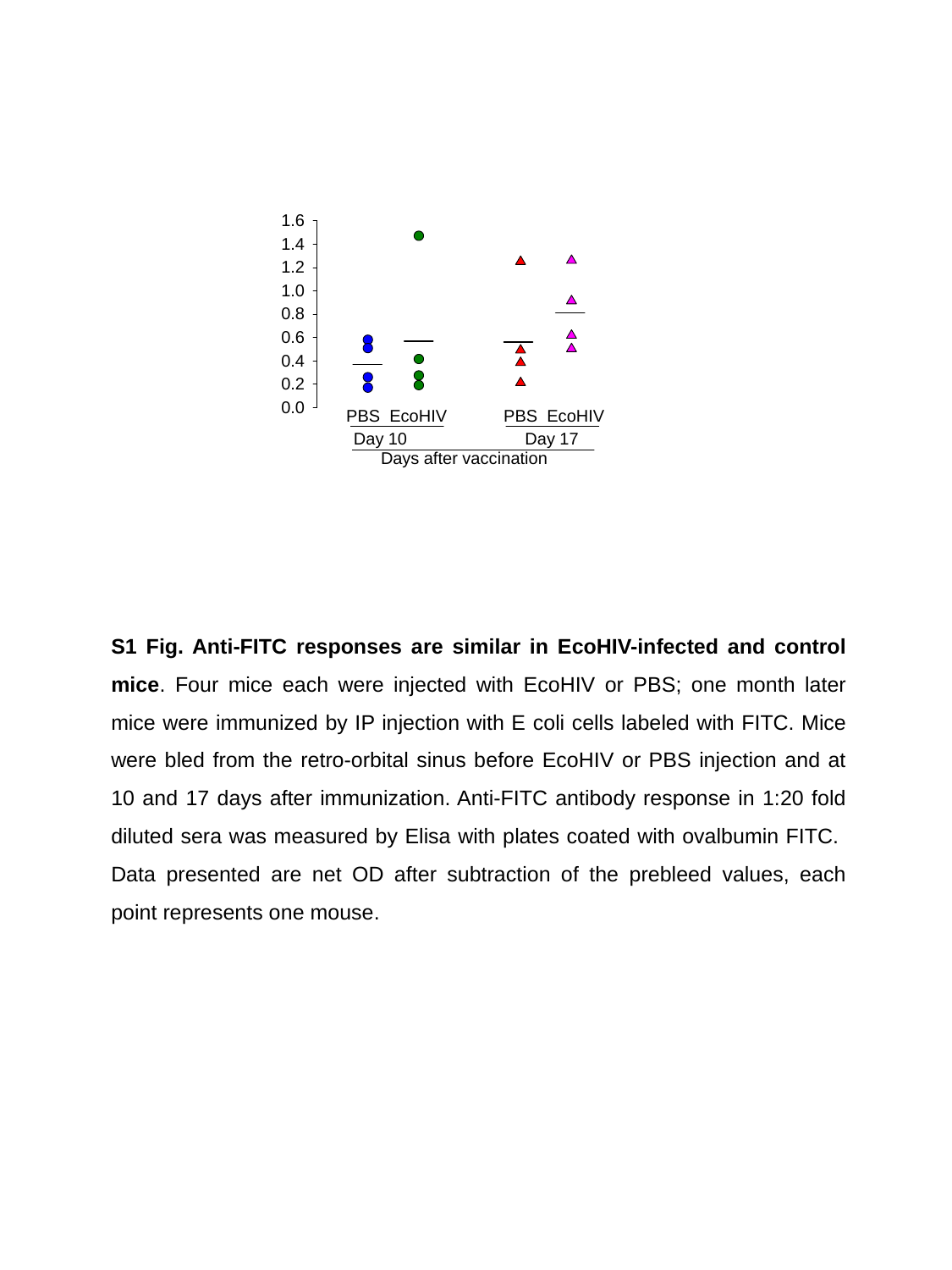

S1 Fig. Anti-FITC responses are similar in EcoHIV-infected and control mice. Four mice each were injected with EcoHIV or PBS; one month later mice were immunized by IP injection with E coli cells labeled with FITC. Mice were bled from the retro-orbital sinus before EcoHIV or PBS injection and at 10 and 17 days after immunization. Anti-FITC antibody response in 1:20 fold diluted sera was measured by Elisa with plates coated with ovalbumin FITC. Data presented are net OD after subtraction of the prebleed values, each point represents one mouse.
