# Supplementary material for: EcoHIV infection of mice establishes latent viral reservoirs in T cells and active viral reservoirs in macrophages that are sufficient for induction of neurocognitive impairment
Source: PLoS Pathog. 2018 Jun 7;14(6):e1007061. doi: 10.1371/journal.ppat.1007061 (PMC5991655; doi:10.1371/journal.ppat.1007061)
Supplement: S2 Fig — (A). CD4+ T cell population were negatively isolated from splenocytes and analyzed by flow cytometer. (B). Peritoneal cells were isolated and cultured in DMEM with 20 ng/ml M-CSF for 6 h and the adherent cells were harvested by Cellstripper and analyzed by flow cytometer. Numbers indicate the percentage of gated cells. (C) Resting CD4+ T lymphocytes were further isolated from total CD4+ T cells and identified by flow cytometer using anti-CD69, anti-CD25. (PPTX) [file ppat.1007061.s002.pptx]

## Slide 1
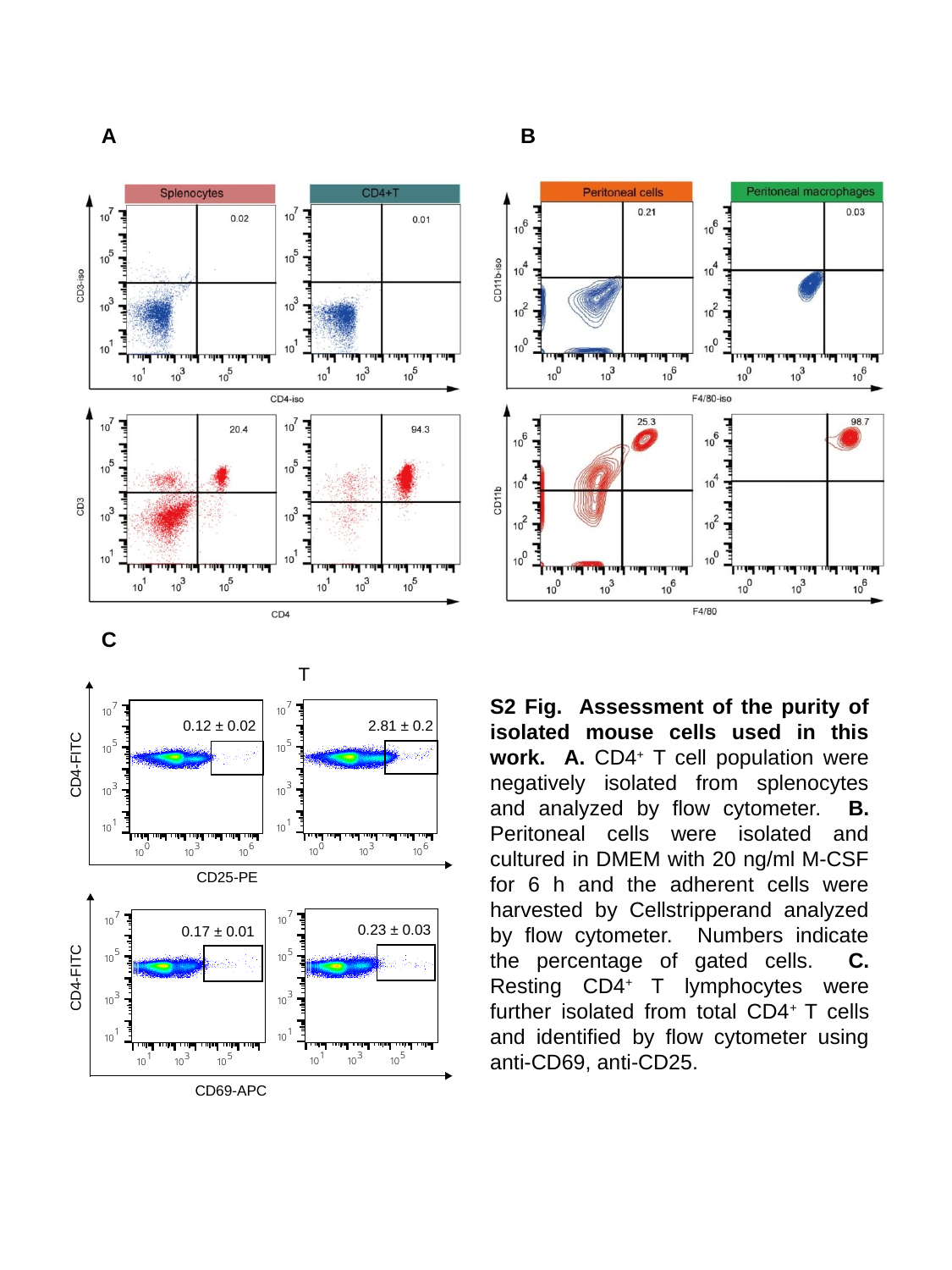

A
B
C
S2 Fig. Assessment of the purity of isolated mouse cells used in this work. A. CD4+ T cell population were negatively isolated from splenocytes and analyzed by flow cytometer. B. Peritoneal cells were isolated and cultured in DMEM with 20 ng/ml M-CSF for 6 h and the adherent cells were harvested by Cellstripperand analyzed by flow cytometer. Numbers indicate the percentage of gated cells. C. Resting CD4+ T lymphocytes were further isolated from total CD4+ T cells and identified by flow cytometer using anti-CD69, anti-CD25.
