# Supplementary material for: EcoHIV infection of mice establishes latent viral reservoirs in T cells and active viral reservoirs in macrophages that are sufficient for induction of neurocognitive impairment
Source: PLoS Pathog. 2018 Jun 7;14(6):e1007061. doi: 10.1371/journal.ppat.1007061 (PMC5991655; doi:10.1371/journal.ppat.1007061)
Supplement: S3 Fig — A. In panels left to right total HIV DNA was measured by QPCR, integrated DNA was measured with nested QPCR, and genomic vRNA was measured by QPCR in PBMC from HIV patients with average CD4+ T cell counts more than 500/μl blood. The line represents the mean value. B. The ratio of integrated to total vDNA for each patient sample or each mouse sample more than 2 months after infection (Fig 2A) and then the mean ratios of groups were obtained. C.D. At 6 weeks after EcoHIV infection, mice were treated with vehicle or abacavir and raltegravir for 14 days prior to tissue collection. Integrated EcoHIV DNA was measured in spleen (C.) or PC (D.). The horizontal bar represents the median of these values. (PPTX) [file ppat.1007061.s003.pptx]

## Slide 1
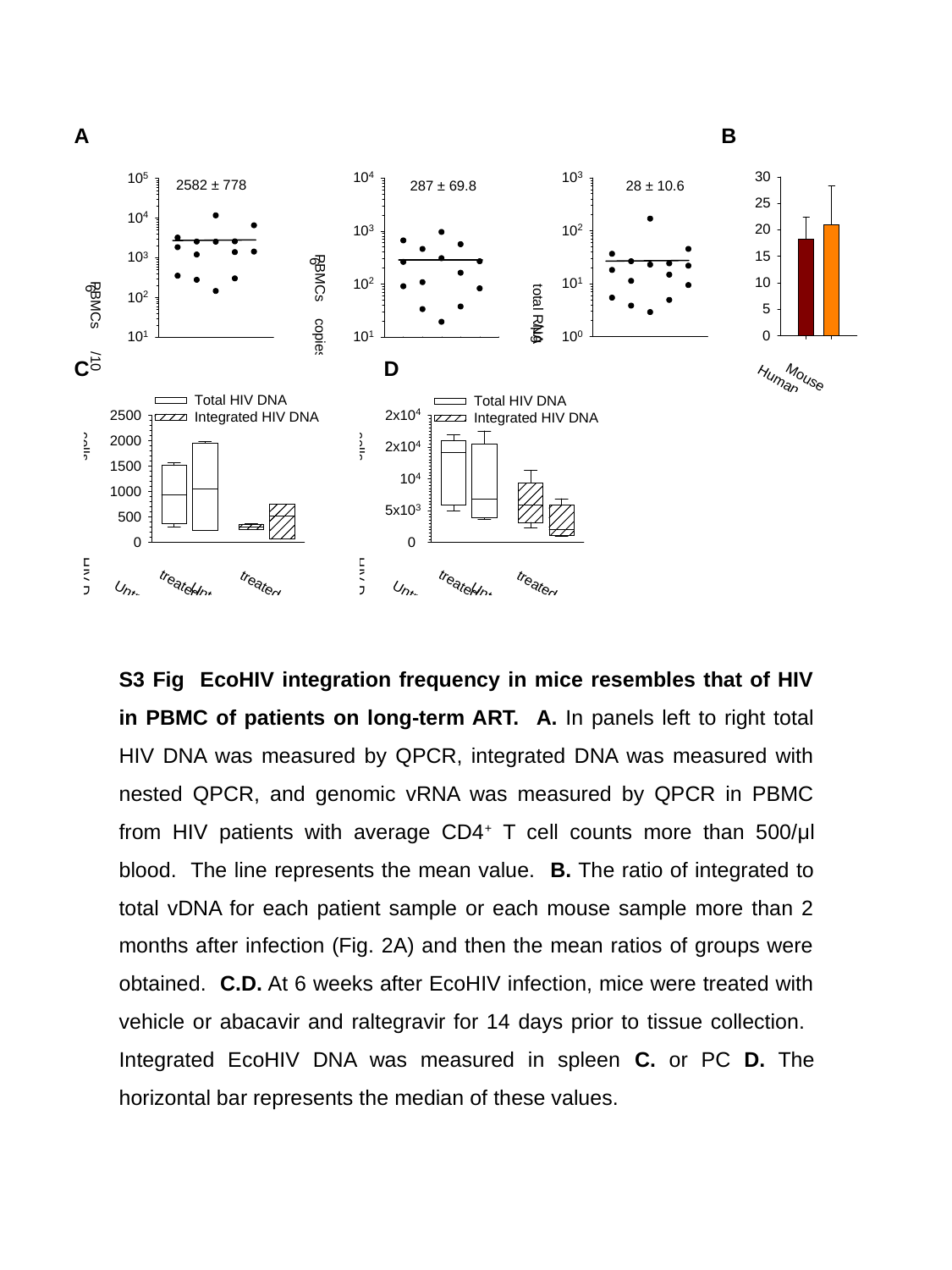

A
B
C
D
S3 Fig EcoHIV integration frequency in mice resembles that of HIV in PBMC of patients on long-term ART. A. In panels left to right total HIV DNA was measured by QPCR, integrated DNA was measured with nested QPCR, and genomic vRNA was measured by QPCR in PBMC from HIV patients with average CD4+ T cell counts more than 500/μl blood. The line represents the mean value. B. The ratio of integrated to total vDNA for each patient sample or each mouse sample more than 2 months after infection (Fig. 2A) and then the mean ratios of groups were obtained. C.D. At 6 weeks after EcoHIV infection, mice were treated with vehicle or abacavir and raltegravir for 14 days prior to tissue collection. Integrated EcoHIV DNA was measured in spleen C. or PC D. The horizontal bar represents the median of these values.
