# Supplementary material for: EcoHIV infection of mice establishes latent viral reservoirs in T cells and active viral reservoirs in macrophages that are sufficient for induction of neurocognitive impairment
Source: PLoS Pathog. 2018 Jun 7;14(6):e1007061. doi: 10.1371/journal.ppat.1007061 (PMC5991655; doi:10.1371/journal.ppat.1007061)
Supplement: S4 Fig — (A-D). Ten days after EcoHIV or MLV infection of mice, the indicated tissues were harvested for measurement of viral nucleic acids by QPCR. (A) 2LTR circular DNA, (B) integrated viral DNA, (D) ENV RNA and (E) Spliced vif RNA. (E) At 7 d after EcoHIV-EGFP or MLV-EGFP infection of mice, peritoneal cells were analyzed for F4/80 and intracellular EGFP expression. Numbers in the flow plots indicates the percentage of gated cells expressing EGFP. Red histograms are isotype controls. BM = bone marrow, SP = spleen, PC = peritoneal cells, LN = lymph nodes, TH = thymus; LI = liver, LU = lung. (PPTX) [file ppat.1007061.s004.pptx]

## Slide 1
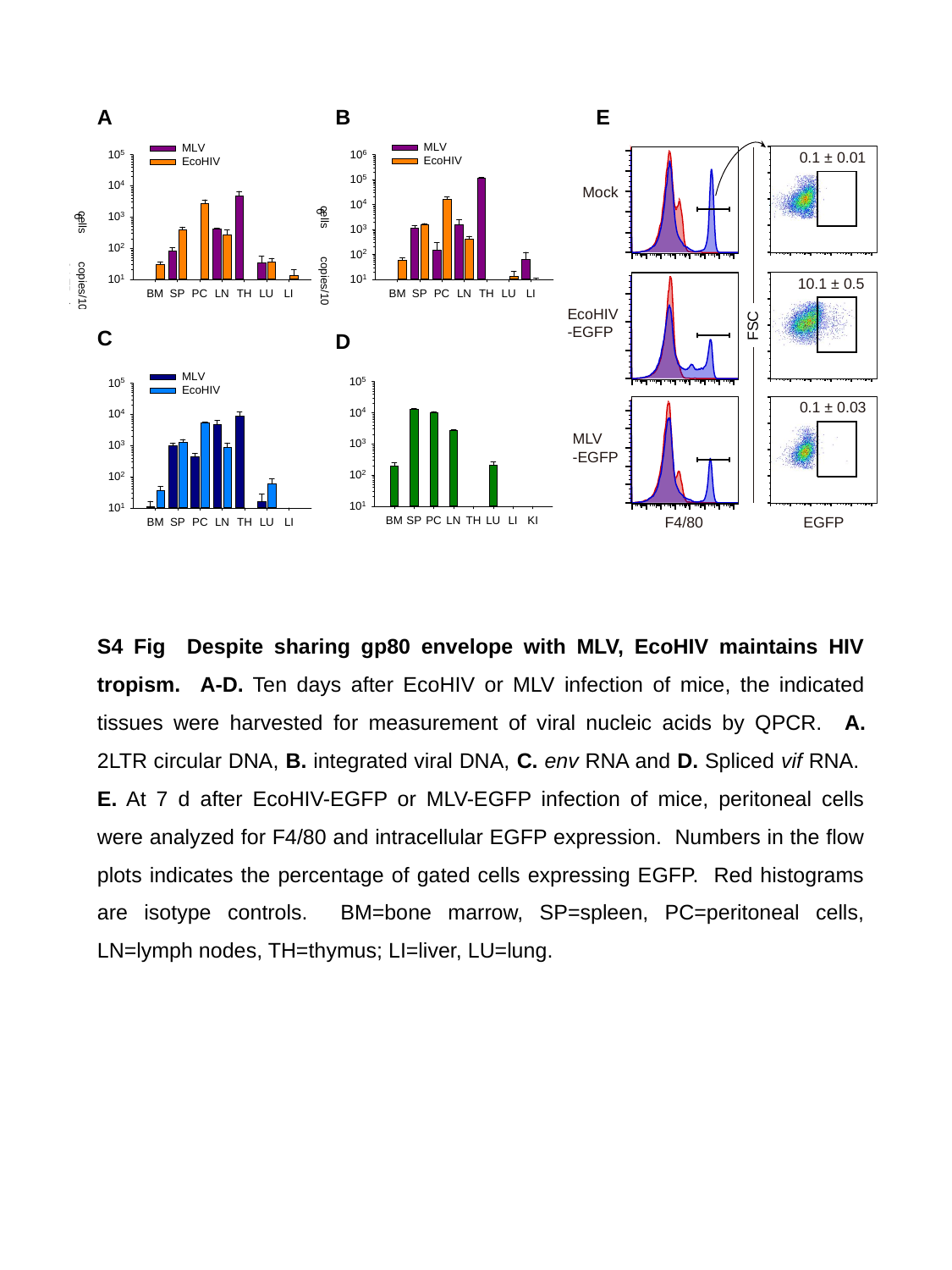

A
B
E
C
D
S4 Fig Despite sharing gp80 envelope with MLV, EcoHIV maintains HIV tropism. A-D. Ten days after EcoHIV or MLV infection of mice, the indicated tissues were harvested for measurement of viral nucleic acids by QPCR. A. 2LTR circular DNA, B. integrated viral DNA, C. env RNA and D. Spliced vif RNA. E. At 7 d after EcoHIV-EGFP or MLV-EGFP infection of mice, peritoneal cells were analyzed for F4/80 and intracellular EGFP expression. Numbers in the flow plots indicates the percentage of gated cells expressing EGFP. Red histograms are isotype controls. BM=bone marrow, SP=spleen, PC=peritoneal cells, LN=lymph nodes, TH=thymus; LI=liver, LU=lung.
