# Supplementary material for: EcoHIV infection of mice establishes latent viral reservoirs in T cells and active viral reservoirs in macrophages that are sufficient for induction of neurocognitive impairment
Source: PLoS Pathog. 2018 Jun 7;14(6):e1007061. doi: 10.1371/journal.ppat.1007061 (PMC5991655; doi:10.1371/journal.ppat.1007061)
Supplement: S5 Fig — (A-D). The genomes of EcoHIV/NDK (A) and variants in (B), (C) and (D) are derived from the molecular clone HIV-1/NDK [127], in which the HIV genes are shown in blue and the MLV gp80 was shown in red and black (deletion). The internal ribosome entry site (IRES) shown in (B) and (C) permits expression of EGFP and luciferase from the HIV RNA transcript. (D) Was constructed by introducing two stop codes followed ATG of the coding region of signal peptide based on (A). E-G. The genomes of MLV variants in (G) was derived from (F) by introducing two stop codes followed ATG of the coding region of signal peptide in gp80. 2A peptide in (F) and (G) was derived from porcine teschovirus-1. (PPTX) [file ppat.1007061.s005.pptx]

## Slide 1
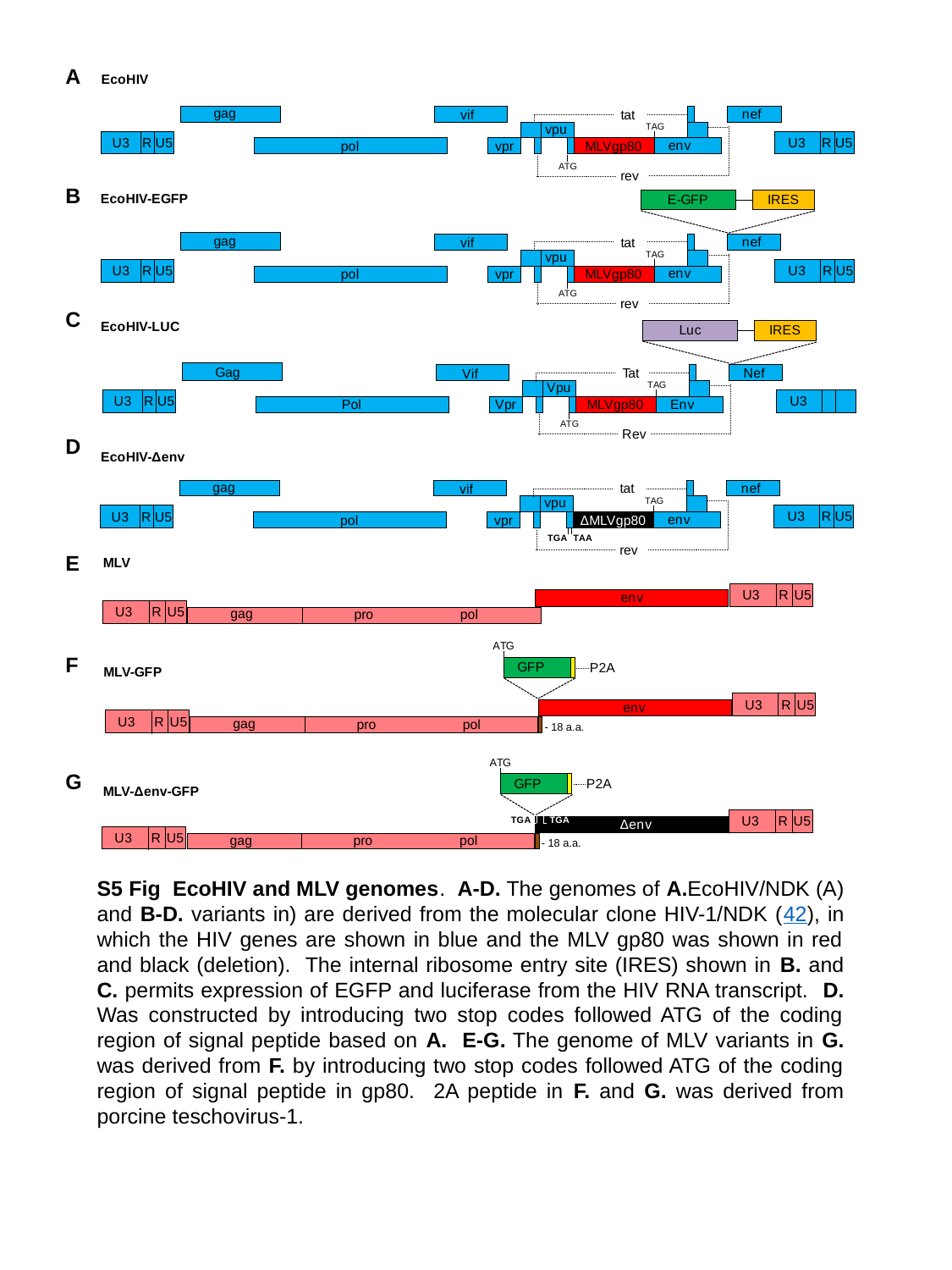

A
B
C
D
E
F
G
S5 Fig EcoHIV and MLV genomes. A-D. The genomes of A.EcoHIV/NDK (A) and B-D. variants in) are derived from the molecular clone HIV-1/NDK (42), in which the HIV genes are shown in blue and the MLV gp80 was shown in red and black (deletion). The internal ribosome entry site (IRES) shown in B. and C. permits expression of EGFP and luciferase from the HIV RNA transcript. D. Was constructed by introducing two stop codes followed ATG of the coding region of signal peptide based on A. E-G. The genome of MLV variants in G. was derived from F. by introducing two stop codes followed ATG of the coding region of signal peptide in gp80. 2A peptide in F. and G. was derived from porcine teschovirus-1.
