# Supplementary material for: EcoHIV infection of mice establishes latent viral reservoirs in T cells and active viral reservoirs in macrophages that are sufficient for induction of neurocognitive impairment
Source: PLoS Pathog. 2018 Jun 7;14(6):e1007061. doi: 10.1371/journal.ppat.1007061 (PMC5991655; doi:10.1371/journal.ppat.1007061)
Supplement: S1 Table — (PPTX) [file ppat.1007061.s006.pptx]

## Slide 1
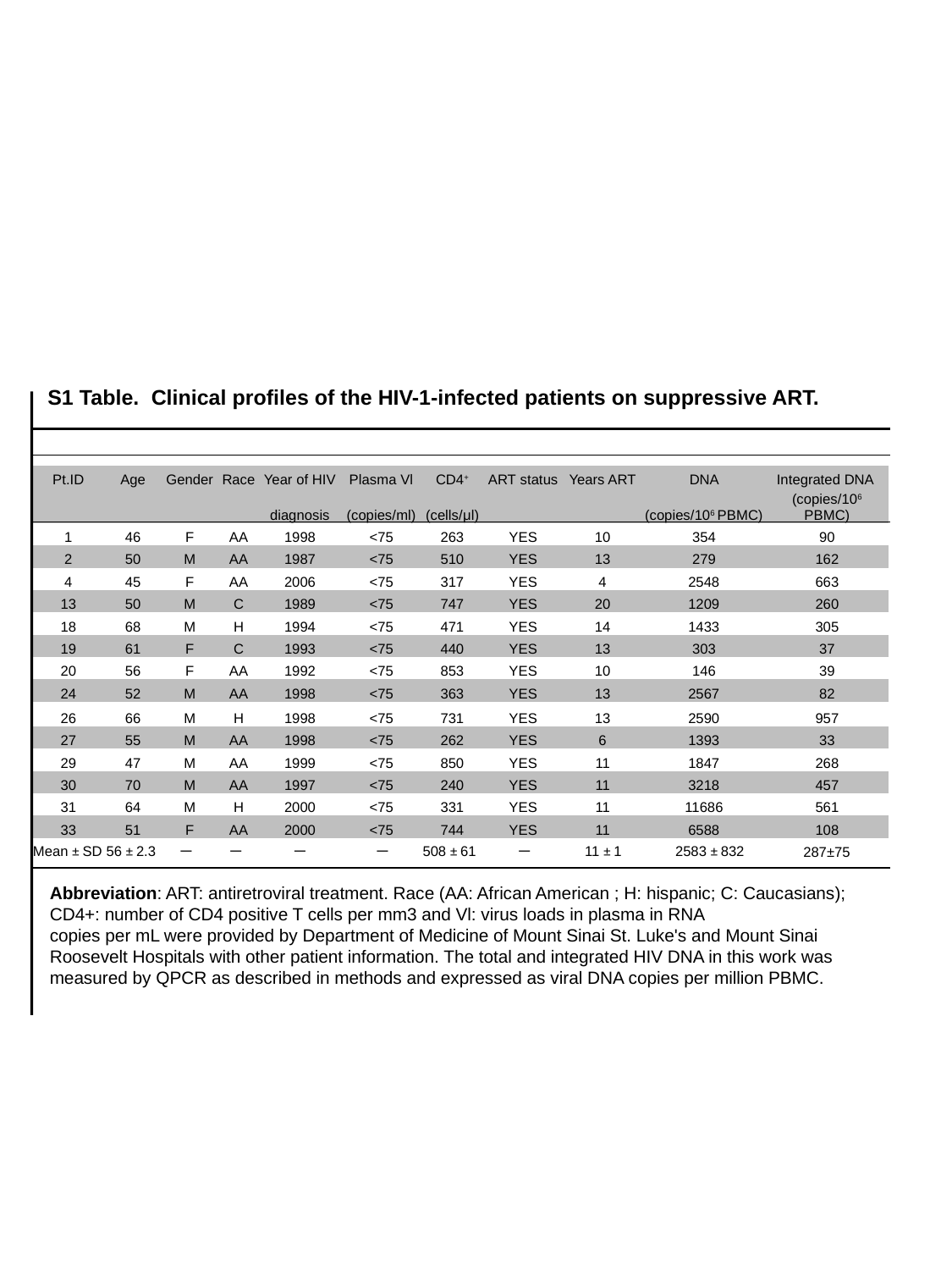

S1 Table. Clinical profiles of the HIV-1-infected patients on suppressive ART.
| Pt.ID | Age | Gender | Race | Year of HIV | Plasma Vl | CD4+ | ART status | Years ART | DNA | Integrated DNA |
| --- | --- | --- | --- | --- | --- | --- | --- | --- | --- | --- |
| | | | | diagnosis | (copies/ml) | (cells/μl) | | | (copies/106 PBMC) | (copies/106 PBMC) |
| 1 | 46 | F | AA | 1998 | <75 | 263 | YES | 10 | 354 | 90 |
| 2 | 50 | M | AA | 1987 | <75 | 510 | YES | 13 | 279 | 162 |
| 4 | 45 | F | AA | 2006 | <75 | 317 | YES | 4 | 2548 | 663 |
| 13 | 50 | M | C | 1989 | <75 | 747 | YES | 20 | 1209 | 260 |
| 18 | 68 | M | H | 1994 | <75 | 471 | YES | 14 | 1433 | 305 |
| 19 | 61 | F | C | 1993 | <75 | 440 | YES | 13 | 303 | 37 |
| 20 | 56 | F | AA | 1992 | <75 | 853 | YES | 10 | 146 | 39 |
| 24 | 52 | M | AA | 1998 | <75 | 363 | YES | 13 | 2567 | 82 |
| 26 | 66 | M | H | 1998 | <75 | 731 | YES | 13 | 2590 | 957 |
| 27 | 55 | M | AA | 1998 | <75 | 262 | YES | 6 | 1393 | 33 |
| 29 | 47 | M | AA | 1999 | <75 | 850 | YES | 11 | 1847 | 268 |
| 30 | 70 | M | AA | 1997 | <75 | 240 | YES | 11 | 3218 | 457 |
| 31 | 64 | M | H | 2000 | <75 | 331 | YES | 11 | 11686 | 561 |
| 33 | 51 | F | AA | 2000 | <75 | 744 | YES | 11 | 6588 | 108 |
| Mean ± SD | 56 ± 2.3 | ― | ― | ― | ― | 508 ± 61 | ― | 11 ± 1 | 2583 ± 832 | 287±75 |
Abbreviation: ART: antiretroviral treatment. Race (AA: African American ; H: hispanic; C: Caucasians); CD4+: number of CD4 positive T cells per mm3 and Vl: virus loads in plasma in RNA
copies per mL were provided by Department of Medicine of Mount Sinai St. Luke's and Mount Sinai Roosevelt Hospitals with other patient information. The total and integrated HIV DNA in this work was measured by QPCR as described in methods and expressed as viral DNA copies per million PBMC.
