# Supplementary material for: EcoHIV infection of mice establishes latent viral reservoirs in T cells and active viral reservoirs in macrophages that are sufficient for induction of neurocognitive impairment
Source: PLoS Pathog. 2018 Jun 7;14(6):e1007061. doi: 10.1371/journal.ppat.1007061 (PMC5991655; doi:10.1371/journal.ppat.1007061)
Supplement: S2 Table — For L-ART pharmacokinetics, plasma samples and brain tissues were collected as indicated and tested by ultra-performance liquid chromatography tandem mass spectrometry for drug concentrations. The samples were from experiment depicted in Fig 8; 3–4 mice were sampled per collection time. (PPTX) [file ppat.1007061.s007.pptx]

## Slide 1
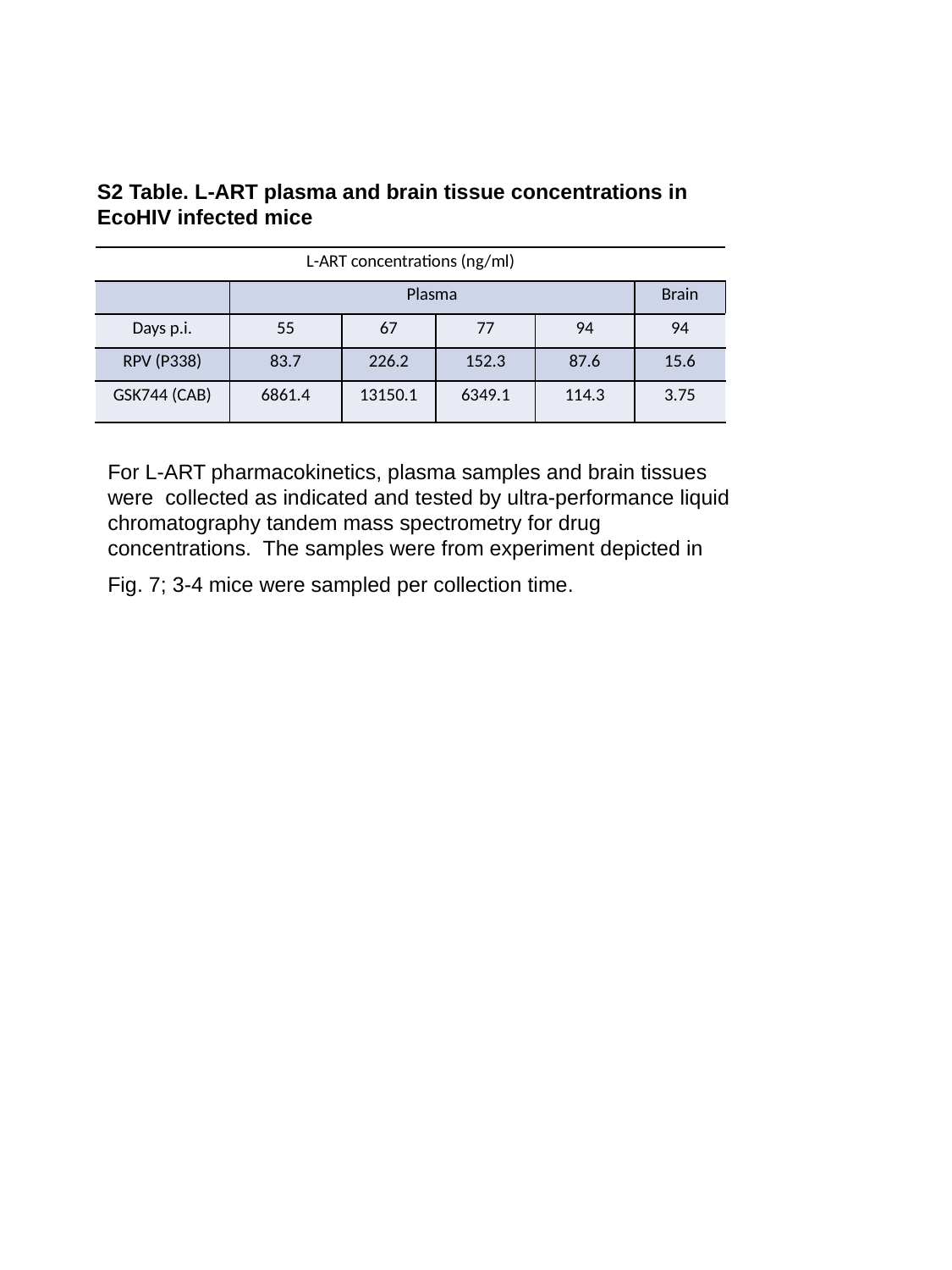

S2 Table. L-ART plasma and brain tissue concentrations in EcoHIV infected mice
| L-ART concentrations (ng/ml) | | | | | |
| --- | --- | --- | --- | --- | --- |
| | Plasma | | | | Brain |
| Days p.i. | 55 | 67 | 77 | 94 | 94 |
| RPV (P338) | 83.7 | 226.2 | 152.3 | 87.6 | 15.6 |
| GSK744 (CAB) | 6861.4 | 13150.1 | 6349.1 | 114.3 | 3.75 |
For L-ART pharmacokinetics, plasma samples and brain tissues were collected as indicated and tested by ultra-performance liquid chromatography tandem mass spectrometry for drug concentrations. The samples were from experiment depicted in Fig. 7; 3-4 mice were sampled per collection time.
